# NMNAT1 Activates Autophagy to Delay D‐Galactose‐Induced Aging in Cochlear Hair Cells

**DOI:** 10.1111/acel.70373

**Published:** 2026-01-11

**Authors:** Yongjie Wei, Wenqing Yang, Han Wu, Mengdie Kong, Dachuan Fan, Yuhua Zhang, Nan Cheng, Jiawei Du, Lingna Guo, Yuyang Li, Ye Zhang, Qian Dai, Wei Cao, Jianming Yang, Qiaojun Fang

**Affiliations:** ^1^ Department of Otolaryngology‐Head and Neck Surgery The Second Affiliated Hospital of Anhui Medical University Hefei China; ^2^ School of Life Science and Technology Southeast University Nanjing China; ^3^ Department of Anesthesiology and Perioperative Medicine The Second Affiliated Hospital of Anhui Medical University Hefei China; ^4^ School of Life Sciences Anhui Medical University Hefei China

**Keywords:** age‐related hearing loss, autophagy, cochlear hair cell, NMNAT1, TCA cycle metabolism

## Abstract

With an aging population, the incidence of age‐related hearing loss (ARHL) continues to increase. Aging cells exhibit reduced nicotinamide adenine dinucleotide (NAD^+^) levels and impaired autophagy; however, the mechanisms underlying these processes remain largely unclear. In our study, we assessed the role of nicotinamide nucleotide adenylate transferase 1 (NMNAT1) in cochlear hair cell aging using D‐galactose (D‐gal)‐induced aging HEI‐OC1 cells and cochlear explants. We observed a significant reduction in NMNAT1 expression in HEI‐OC1 cells and cochlear hair cells treated with D‐gal. Notably, NMNAT1 overexpression activated autophagy and decelerated hair cell aging. Metabolomic analysis revealed a dysregulated tricarboxylic acid cycle in *Nmnat1*‐knockout cells, indicating that NMNAT1 regulates autophagy and metabolic pathways that affect hair cell aging. These findings offer novel insights into the association between autophagy and metabolism during aging and highlight NMNAT1 as a potential therapeutic target for the prevention and treatment of ARHL.

## Graphical abstract

The study presents mechanistic insights into cochlear hair cells aging using cellular and explant models. The assessment into NMNAT1 regulated NAD^+^ metabolism, autophagy, and aging in ARHL. NMNAT1 overexpression restores autophagic activity and decelerates aging in cochlear hair cells.

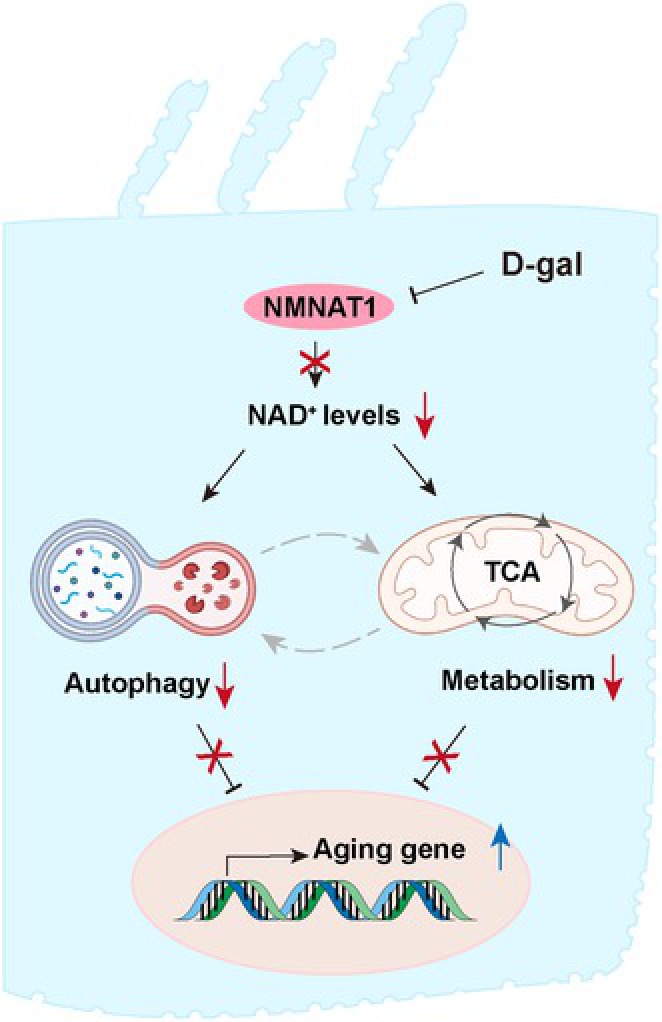

## 1 Introduction

With an aging population, the incidence of age‐related hearing loss (ARHL) continues to increase (Dzau and Balatbat 2018). Among these, age‐related hearing loss (ARHL) is the most prevalent sensory disturbance affecting the aged. Clinically, ARHL typically presents as bilateral symmetrical hearing loss that begins at high sound frequencies and gradually progresses to medium and low frequencies (Davalli et al. 2016). As its incidence increases with age, ARHL has become the predominant cause of hearing disability among aged. According to the World Hearing Report, over 65% of individuals aged ≥ 60 years exhibited a certain degree of hearing loss (WHO 2021). This impairment can result in significant social isolation, communication challenges, depression, and even cognitive decline in older adults (Jafari et al. 2021; Uchida et al. 2019). The pathogenesis of ARHL remains inadequately understood, underscoring the urgent need for further research to elucidate the potential mechanisms and enhance clinical diagnosis and treatment.

ARHL is a chronic, age‐associated condition that is caused by cumulative effects of harmful lesions and dysfunction within the auditory system (Eckert et al. 2021). The cochlea is the primary site of ARHL‐related pathology, with characteristic alterations, including damage to hair cells and spiral ganglion neurons (Youn et al. 2020; Tawfik et al. 2020). Reduced cochlear vascularization, DNA damage, ROS accumulation, and mitochondrial dysfunction are the primary contributors to cochlear lesions (Bazard et al. 2021; Kociszewska and Vlajkovic 2022; Yang et al. 2023). With aging, diminished cellular metabolism and self‐repair capabilities cause damaged organelles and misfolded proteins to accumulate (Ye et al. 2018). Nicotinamide adenine dinucleotide (NAD) is a cofactor for maintaining enzyme function associated with energy metabolism and mitochondrial stability (Wu et al. 2024; Navas and Carnero 2021; Lautrup et al. 2019). Notably, NAD^+^ levels decline with age (McReynolds et al. 2020; Covarrubias et al. 2021). Both total NAD^+^ levels and the NAD^+^/NADH ratio were significantly reduced in the hair cells of aged mice. Short‐ and long‐term NAD^+^ supplementation in these mice enhances synaptic connectivity between hair cells and auditory neurons, thereby mitigating ARHL (Okur et al. 2020, 2023).

Macroautophagy (hereafter referred to as autophagy) is specifically crucial for preserving protein stability, organelle integrity, and overall cellular health and viability (Debnath et al. 2023; Levine and Kroemer 2019). Autophagy declines in aging cells, coinciding with reduced NAD^+^ levels—increasing the NAD^+^ pool or enhancing autophagy alone helps slow the aging process (Bjedov et al. 2020; Mitchell et al. 2018). NAD^+^ plays a crucial role in regulating cellular autophagy. Sirtuin 1 (SIRT1) activates various autophagy‐related proteins through NAD^+^‐dependent histone deacetylation (Yu et al. 2024). Pang et al. (2019) reported that SIRT1 expression and autophagy were reduced in aged mice hair cells. By activating SIRT1, autophagy is enhanced by deacetylating autophagy‐related protein 9A, an autophagy‐related protein—that consequently mitigates age‐related hair cell loss (Xiong et al. 2019; Pang et al. 2019). However, the mechanisms underlying autophagy regulation in ARHL require further assessment.

Nicotinamide nucleotide adenylate transferase 1 (NMNAT1) is a key enzyme in the biosynthesis of NAD^+^, maintaining intracellular NAD^+^ levels and NAD+/NADH ratio (Huang et al. 2024; Shi et al. 2021; Sokolov et al. 2021). In Werner syndrome, the transcriptional suppression of *Nmnat1* reduces NAD^+^ levels, thereby accelerating aging. NAD^+^ supplementation significantly alleviates accelerated aging (Fang et al. 2019). During hypoxic stress, NMNAT1 overexpression enhances autophagy and protects neuronal dendrites from hypoxia‐induced damage (Wen et al. 2013). Additionally, NMNAT1 activation prevents acute ischemic stroke in aging rats by modulating the SIRT1/mTOR pathway that induces autophagy during focal cerebral ischemia (Wang et al. 2019). In Alzheimer's disease (AD), NMNAT1 overexpression facilitates amyloid clearance through autophagy activation (Zhu et al. 2022). However, the specific role of NMNAT1 in ARHL remains unclear.

In our study, we assessed the regulatory relationship between NMNAT1 and autophagy in ARHL mice. D‐galactose (D‐gal) was used to induce aging in HEI‐OC1 (House Ear Institute‐Organ of Corti 1) cells and cochlear explants to establish ARHL model, as previously reported (He, Li, et al. 2021; Zhao et al. 2022). Our results demonstrated that NAD^+^ and autophagy levels were markedly reduced in the D‐gal‐induced ARHL model. NMNAT1 overexpression restores autophagic activity and decelerates aging in cochlear hair cells. Our results offer novel insights into the relationship between NMNAT1, autophagy, and aging, revealing NMNAT1 as a promising target for the clinical management of ARHL.

## 2 Materials and Methods

### 2.1 Animals

Friend Virus B‐type (FVB) mice were purchased from SiPeiFu Biotechnology (Beijing, China) and maintained at the Core for Laboratory Animal Medicine, Institute of Health and Medicine, Hefei Comprehensive National Science Center, China. Postnatal day 3 (P3) FVB mice were used for cochlear explant culture. FVB mice were selected based on their well‐defined genetic background, high fecundity, and large litter size, ensuring a consistent and sufficient supply of experimental animals. The cochleae were dissected and immediately placed in precooled Hank's balanced salt solution (HBSS, 14175095; Gibco). The cochlear volute was carefully removed under a dissecting microscope using forceps, and the basement membrane along with the spiral ligament was gently isolated. Subsequently, the basement membrane was separated from the spiral ligament, adhered to an 8 mm glass slide pre‐coated with Cell‐Tak (354240; Corning), and transferred into a 4‐well culture dish. Each well contained 2 mL Dulbecco's modified Eagle's medium (DMEM) (C11995500BT; Gibco) supplemented with 1% N2 (A1370701; Gibco), 2% B27 (17504044; Gibco), and 50 μg/mL ampicillin (A0166; Sigma‐Aldrich). The cultures were incubated at 37°C with 5% CO_2_. All animal experiments were conducted in strict accordance with the ethical guidelines for laboratory animals established by the Anhui Medical University, China.

### 2.2 Cell Culture

HEI‐OC1 cells (M8‐0401) were obtained from Cyagen (Guangzhou, China). Cells were maintained in DMEM supplemented with 10% fetal bovine serum (FBS) and 50 μg/mL ampicillin at 33°C in a 10% CO_2_ incubator. When the cells were approximately 90% confluence, they were digested into single‐cell suspensions using 0.25% trypsin‐ethylenediaminetetraacetic acid (EDTA) (25200054; Thermo Fisher Scientific) for passaging and subsequent experiments.

### 2.3 Cell Counting Kit‐8 Assay

HEI‐OC1 cells were plated in 96‐well plates (100 μL per well) and cultured for 12–24 h. Once the cell density reached 60%–70%, they were treated with DMEM containing 2, 5, 10, 20, and 40 mg/mL D‐gal (G7050; Sigma‐Aldrich) for 72 h. Subsequently, 100 μL DMEM supplemented with 10% Cell Counting Kit‐8 solution (HY‐K0301; MCE) was added to each well. After incubation at 37°C for 2 h, the absorbance at 450 nm was measured using a microplate reader (Bio‐Rad). Cell viability was calculated using the formula: Cell viability (%) = ([As−Ab]/[Ac − Ab]) × 100%—where As represents the absorbance of the experimental wells (cells, culture medium and CCK‐8 solution, with drugs); Ac represents the absorbance of control wells (cells, culture medium and CCK‐8 solution, without drugs); and Ab represents the absorbance of blank wells (medium, CCK‐8 solution, without cells and drugs).

### 2.4 Mito‐SOX/DCFH‐DA Staining Analysis

The MitoBright ROS Deep Red‐Mitochondrial Superoxide Detection Kit (MT16; DOJINDO) and ROS Assay Kit‐Highly Sensitive DCFH‐DA (R252; DOJINDO) were purchased from DOJINDO. HEI‐OC1 cells were treated with D‐gal (5 and 20 mg/mL) for 72 h. After treatment, the culture medium was removed, and the cells were washed twice with phosphate‐buffered saline (PBS). Following incubation with Mito‐SOX or DCFH‐DA reagent at 37°C for 30 min. Subsequently, the cells were mounted on slides for confocal imaging (LSM900; ZEISS).

### 2.5 Flow Cytometry Analysis

HEI‐OC1 cells were treated with D‐gal (5 and 20 mg/mL) for 72 h. The cells were harvested using trypsin (15090046; Gibco), incubated with Mito‐SOX or DCFH‐DA reagent at 37°C for 30 min. Then, the cells were resuspended with PBS for flow cytometry analysis.

### 2.6 Western Blotting

Proteins were extracted from HEI‐OC1 cells and cochlear explants using RIPA buffer (P0013B; Beyotime) supplemented with a protease inhibitor cocktail (04693132001; Roche, Basel, Switzerland). Protein concentration was estimated using the bicinchoninic acid Enhanced Protein Assay Kit (P0010; Beyotime). The primary antibodies used in this study were as follows: rabbit anti‐β‐tubulin (1:40000, 5568; CST), rabbit anti‐β‐Actin (1:40000, 20536‐1‐AP; Proteintech), mouse anti‐GAPDH (1:4000, ab8245; Abcam), rabbit anti‐senescence marker protein‐30 (SMP30) (1:1000, 17947‐1‐AP; Proteintech), rabbit anti‐lamin B1 (1:1000, 13435; CST), rabbit anti‐γ‐H2A.X (1:400, 2577; CST), rabbit anti‐P21 (1:1000, 37543; CST), rabbit anti‐P16 (1:1000, 29271; CST), rabbit anti‐NMNAT1 (1:1000, 98354; CST), rabbit anti‐NMNAT1 (1:400, 11399‐1‐AP; Proteintech), rabbit anti‐LC3B (1:1000, 3868; CST), rabbit anti‐P62 (1:1000, 23214; CST), mouse anti‐autophagy related 7 (ATG7) (1:1000, 67341; Proteintech), and rabbit anti‐BECLIN1 (1:1000, 3495; CST). The secondary antibodies used were anti‐rabbit IgG (1:4000, CST; 7074) and anti‐mouse IgG (1:4000, CST; 7076). Protein bands were visualized using the Tanon Gel Imaging System (Tanon, China), and quantified using ImageJ software, with tubulin or actin serving as an internal reference.

### 2.7 Immunostaining

The cells or cochlear explants were fixed using 4% paraformaldehyde (P6148; Sigma‐Aldrich). The samples were permeabilized by incubation in PBST (PBS containing 1% Triton X‐100 (1231O021; Solarbio)) for 30 min, and blocking with 10% FBS (SL050; Solarbio) for 1 h, and incubated overnight at 4°C with appropriately diluted primary antibodies. After three 10‐min washes with PBST, the samples were incubated for 2 h at RT with appropriately diluted secondary antibodies. Nuclei were stained using DAPI (1:1000, C0065; Solarbio) before mounting. The primary antibodies used were as follows: rabbit anti‐NMNAT1 (1:400, 11399‐1‐AP; Proteintech), rabbit anti‐myosin7a (1:1000, 25‐6790; Proteus Bioscience), mouse anti‐myosin7a (1:400, 138‐1; DSHB), and rabbit anti‐γ‐H2A.X (1:400, 2577; CST). The secondary antibodies used were Alexa Fluor 555 donkey anti‐rabbit (1:400, Invitrogen, A‐31572), Alexa Fluor 488 donkey anti‐rabbit (1:400, Invitrogen, A‐21206), and Alexa Fluor 488 donkey anti‐mouse (1:400, Invitrogen, R37114). Fluorescence images were photographed using a confocal microscope (LSM900; ZEISS).

### 2.8 Semi‐Quantification of the Immunolabeling Signals From Outer Hair Cells (OHCs)

Semi‐quantification of immunolabeled signals is a well‐accepted method (He, Pan, et al. 2021; Yuan et al. 2015). NMNAT1 immunolabeling in OHCs was semi‐quantified from confocal images captured with a × 63 lens under equal parameter settings. The fluorescence linear ranges of NMNAT1 were semi‐quantified using the ImageJ software (National Institutes of Health, Bethesda, MD, USA). Individual OHCs were outlined using the ImageJ circle tool based on myosin7a staining. NMNAT1 fluorescence in grayscale OHCs was measured, and background was subtracted. The average grayscale intensity of the NMNAT1 fluorescence was calculated for each OHC. The relative grayscale intensity was normalized to that of the control group.

### 2.9 Senescence‐Associated β‐Galactosidase (SA‐β‐Gal) Staining

Cellular Senescence β‐Galactosidase Staining Kit (C0602) was purchased from Beyotime (China). Cells and cochlear explants were exposed to D‐gal for 72 h, followed by removal of the culture medium and washing with PBS. Subsequently, added 1 mL β‐galactosidase staining fixative for 15 min at RT. The cells were aspirated and washed thrice with PBS or HBSS for 3 min each. Subsequently, 1 mL of staining solution was added, and the samples were incubated overnight at 37°C.

### 2.10 NAD ^+^/NADH Measurements

NAD^+^/NADH levels were measured using an Enhanced NAD^+^/NADH Assay Kit (S0176S; Beyotime). The cell culture medium was aspirated and discarded, and added 200 μL of NAD^+^/NADH extract buffer per million cells. After cell lysis, the cell supernatant was collected. The samples were diluted 5‐fold with NAD^+^/NADH extract and transferred to a 96‐well plate to determine the total NAD^+^ and NADH levels. A 100 μL aliquot of the diluted sample was incubated at 60°C for 30 min to degrade NAD^+^. Subsequently, 20 μL of the extract buffer was added to measure the NADH levels. The NAD^+^ concentration was calculated as follows: [NAD^+^] = ([NAD^+^] + [NADH]) − [NADH].

### 2.11 Proteomics Analysis

Proteomic analysis was conducted in collaboration with LUMINGBIO (Shanghai, China). For sample preparation, HEI‐OC1 cells were exposed to D‐gal (20 mg/mL) for 72 h. Then, the cells were washed thrice with 10 mL PBS on ice, and after trypsin‐EDTA (25200072; Gibco) digestion, PBS was added to the cell suspension. Following centrifugation (4°C, 1000 × g, 5–10 min), the PBS was removed. The cell pellets were flash‐frozen in liquid nitrogen, and stored at −80°C until shipment on dry ice for proteomic analysis. For proteomic profiling, total proteins were extracted from the samples. An aliquot was reserved for protein quantification and quality control by SDS‐PAGE. The remaining portion was digested with trypsin and labeled using Tandem Mass Tag (TMT) reagents (Thermo Fisher Scientific). Equal amounts of each TMT‐labeled sample were combined and then fractionated by chromatography. The resulting peptides were analyzed by liquid chromatography–tandem mass spectrometry (LC–MS/MS) using an Easy‐nLC 1100 system (Thermo Scientific) coupled to an Orbitrap Fusion Lumos Tribrid mass spectrometer (Thermo Scientific). Raw data were processed with Proteome Discoverer 2.4 (Thermo Fisher Scientific), applying a false discovery rate (FDR) threshold of 0.01 at both the protein and peptide levels. Following quality control and preprocessing, we performed analyses at the expression and functional levels. Specifically, differentially expressed proteins were identified and subsequently subjected to Gene Ontology (GO) enrichment analysis, pathway analysis, and protein–protein interaction (PPI) network analysis. All identified proteins are listed in Table S1.

### 2.12 Nucleoplasmic Separation Assay

Nuclear and cytoplasmic protein extraction kits (Beyotime P0028) were purchased from Beyotime (Shanghai, China). HEI‐OC1 cells were washed with precooled PBS and collected using cell scrapers. Nucleoplasmic separation experiments were performed using the kit, following the manufacturer's protocol.

### 2.13 HBLV‐mCherry‐GFP‐LC3B Cell Line Construction

HBLV‐mCherry‐GFP‐LC3B lentivirus was obtained from HANBIO, China. HEI‐OC1 cells were infected with HBLV‐mCherry‐GFP‐LC3B lentivirus (multiplicity of infection = 10) at a density of 30%–40%. After 48 h, HEI‐OC1 cells were cultured in a medium containing puromycin (5 μg/mL) and screened for at least 48 h. When all the puromycin‐treated untransfected cells in the control group died, all remaining cells in the infected virus group were positive. Subsequently, a stably expressing mCherry‐GFP‐LC3B cell line was established. Autophagic flux was assessed by monitoring mCherry‐GFP‐LC3B expression. Green fluorescence protein (GFP) fluorescence is quenched in acidic environments. When the lysosomes fuse with the autophagosomes to form autolysosomes, GFP is quenched, whereas mCherry remains stable. Yellow puncta (GFP^+^ mCherry^+^) represent autophagosomes. Red puncta (GFP^−^ mCherry^+^) indicate acidic autolysosomes formed after autophagosomes fuse with lysosomes (GFP signal is quenched). Alterations in the number of red spots or the red/yellow spot ratio are reliable indicators of autophagic flux.

### 2.14 Drug Treatment

HEI‐OC1 cells were exposed to 100 nM rapamycin (HY‐10219; MedChemExpress), an autophagy activator, for 12 or 24 h. Autophagy levels were assessed using western blotting. Additionally, autophagy levels were determined after treating the cells with 100 nM bafilomycin A1 (HY‐100558; MedChemExpress), an autophagy inhibitor that targets the late stage of autophagy, for 12 h.

### 2.15 Plasmid and AAV2.7m8‐*Nmnat1* Construction

The recombinant adeno‐associated virus (AAV) vector pAAV‐CMV‐*Nmnat1*‐EGFP plasmid was generated by Tsingke Biotech (Beijing, China). It was transfected into cells by co‐incubating with Lipofectamine 2000 transfection reagent (11668027; Invitrogen). The AAV2.7m8‐Ctrl and AAV2.7m8‐*Nmnat1* (containing pAAV‐CMV‐*Nmnat1*‐3xFLAG‐EF1‐GdGreen‐WPRE) were constructed by Hanbio Biotechnology. After culturing the cochlear explants for 12 h, they were infected with AAV2.7m8‐Ctrl and AAV2.7m8‐*Nmnat1* at a titer of 5 × 10^10^ for 36 h. Subsequently, the explants were exposed to D‐gal for 72 h, followed by immunofluorescence analysis.

### 2.16 *Nmnat1*‐KO Cell Line Construction

The HEI‐OC1‐*Nmnat1*‐KO cell line was generated using CRISPR/Cas9 technology in collaboration with EDITGENE (Guangzhou, China). Based on *Nmnat1* gene sequence data, two single‐guide RNAs (sgRNAs) were designed at the 5′ end of the open reading frame. sgRNA 1 (5′→3′): gaacagcctgaggtgcatgt and sgRNA 2 (5′→3′): tgaggtgcatgttggtgatg. The efficiency of *Nmnat1* knockout (KO) was verified using RT‐qPCR and western blot. The off‐target sites of sgRNA1 and sgRNA2 were predicted using the CCTop‐CRISRP/Cas9 target online prediction website. The design of primers targeting potential off‐target sites and off‐target efficiency was verified using PCR and q‐PCR. The PCR primer sequences for predicting the off‐target sites are as follows: *Prkar1b*‐forward (5′ → 3′): GAGTTGTCCAAAGTATGCAGTAGT, *Prkar1b*‐reverse (5′ → 3′): AGAGTACCTG CCTAGCCTATGT; WD repeat domain 92 (*Wdr92*)‐forward (5′ → 3′): AGGGTGTGGTAGGAGAGACTG, *Wdr92*‐reverse (5′ → 3′): GGCCCAGGCTAAAAACCTTC; basonuclin 2 (*Bnc2*)‐forward (5′ → 3′): AGCATTTCCCTTCCCAATAAGAGA, *Bnc2*‐reverse (5′ → 3′): TGTTCTGATTTGA CCAGAGATTTCG; predicted gene 4117 (*Gm4117*)‐forward (5′ → 3′): GGCCAAACCCTCCCATATCAA, *Gm4117*‐reverse (5′ → 3′): AAGAGAGGCGTGTTTTTCCCA; and *Uhrf2*‐forward (5′ → 3′): GCTAGGTCCACTCAGACACAC; *Uhrf2*‐reverse (5′ → 3′): GGAGAGAG GCTCAGGTCAGA. The q‐PCR primer sequences for predicting the off‐target sites are as follows: *Prkar1b*‐forward (5′ → 3′): GAGTTGTCCAAAGTATGCAGTAGT, *Prkar1b*‐reverse (5′ → 3′): AGAGTACCTG CCTAGCCTATGT; and *Uhrf2*‐forward (5′ → 3′): GCTAGGTCCACTCAGACACAC; *Uhrf2*‐reverse (5′ → 3′): GGAGAGAG GCTCAGGTCAGA.

### 2.17 Untargeted Metabolomics

Metabolomic analysis was conducted in collaboration with LUMINGBIO (Shanghai, China). For sample preparation, added 1 mL mixture (methanol: water, V: V = 4:1) to the samples in two portions and transferred to a glass vial. Subsequently, 200 μL of chloroform was added, followed by ultrasound treatment in an ice bath (500 W; 3 min, 6 s On, 4 s Off). The liquid was aspirated into centrifuge tubes, and subjected to ultrasound in an ice bath for 20 min before being stored overnight at −40°C. The samples were centrifuged at 12,000 × *g* for 10 min at 4°C, and 400 μL of the supernatant was collected, concentrated, and dried using a centrifugal concentrator. Then, 80 μL of methoxamine salt pyridine solution (15 mg/mL) was added, and the mixture was incubated for 60 min at 37°C. Then, equilibrated at RT for 30 min. Chromatographic separation was performed using a DB‐5MS capillary column (30 m × 0.25 mm × 0.25 μm, Agilent J&W Scientific, Folsom, CA, USA). High‐purity helium (≥ 99.999%) was used as the carrier gas at a flow rate of 1.0 mL/min. The injection port temperature is 260°C; injection volume, 1 μL, split less injection; and solvent delay, 5 min. Temperature program: initial column temperature, 60°C (maintained for 0.5 min); increase temperature at 8°C/min to 125°C; then to 210°C, followed by 15°C/min to 270°C; and 20°C/min to 305°C (maintained for 5 min). Mass spectrometric detection was performed using electron impact ion source at 70 eV, with the ion source temperatures at 230°C and quadrupole temperatures at 150°C. Full‐scan acquisition was conducted over the *m/z* range of 50–500. Biologically significant differential metabolites were identified, and the significance of the differences between groups was assessed using variable importance in projection (VIP) values and Student's *t*‐test. Metabolites with *p* value < 0.05 and VIP > 1 were considered indicative of significant differences. The raw data (list) of all individual metabolites in the supplemental information Table S2.

### 2.18 Statistical Analysis

Statistical analyses were performed using GraphPad Prism version 9. Western blot results are presented as the mean ± standard deviation (SD), and group comparisons were analyzed using a one‐sample *t*‐test. Immunofluorescence staining results are presented as the mean ± standard error of the mean. Student's *t*‐test was used to compare the number of γ‐H2A.X aggregation spots and myosin7a^+^ cells. Significance was determined based on the following thresholds: *p < 0.05, **p < 0.01, ***p < 0.001, and ****p < 0.0001, *p* value greater than 0.05 was considered not statistically significant.

## 3 Results

### 3.1 Establishment of Aging Model in HEI‐OC1 Cells and Cochlear Explants Treated With D‐Gal

**FIGURE 1:**
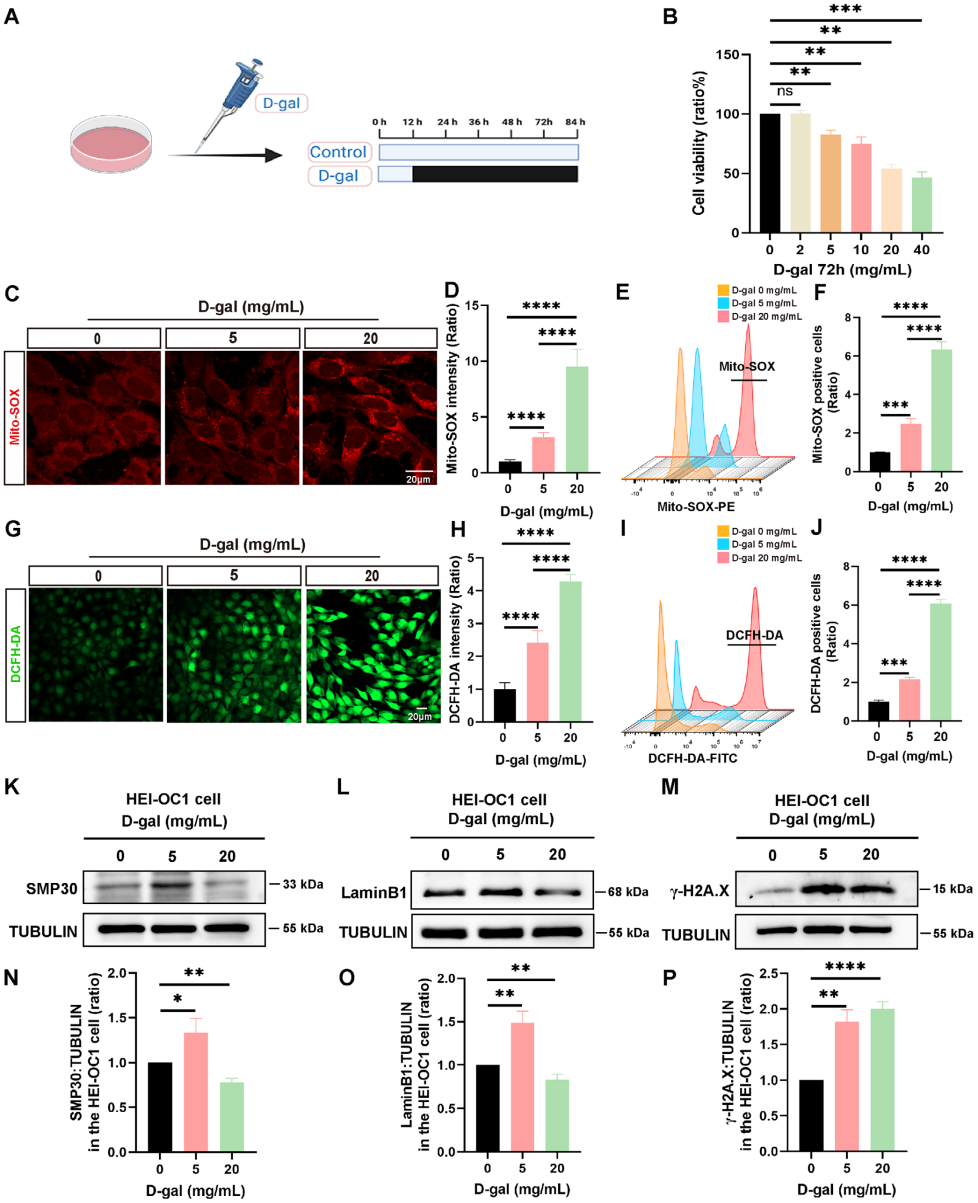
Establishment of D‐gal‐treated aging model in HEI‐OC1 cells. (A) Schematic illustration of D‐gal treatment in HEI‐OC1 cells. (B) Cell viability after exposure to different concentrations of D‐gal, evaluated using the CCK‐8 assay (*n* = 4, independent samples). (C) Immunofluorescent staining of Mito‐SOX in HEI‐OC1 cells exposed to D‐gal (5 and 20 mg/mL) for 72 h. (D) Quantitative analysis of Mito‐SOX intensity in C (*n* = 4, independent samples). (E) Mito‐SOX levels were detected using flow cytometry in HEI‐OC1 cells exposed to D‐gal (5 and 20 mg/mL) for 72 h. (F) Statistical analysis of Mito‐SOX levels in E (*n* = 3, independent samples). (G) Immunofluorescent staining of DCFH‐DA in HEI‐OC1 cells. (H) Quantitative analysis of DCFH‐DA intensity in G (*n* = 4, independent samples). (I) DCFH‐DA levels were detected using flow cytometry in HEI‐OC1 cells. (J) Statistical analysis of DCFH‐DA levels in I (*n* = 3, independent samples). (K–M) Western blot analysis of senescence marker protein‐30 (SMP30), Lamin B1, and γ‐H2A.X in HEI‐OC1 cells treated with D‐gal. (N–P) Statistical analysis of SMP30 (*n* = 4, independent samples), Lamin B1 (*n* = 3, independent samples), and γ‐H2A.X (*n* = 3, independent samples) expression in K–M. Scale bar: 20 μm. Statistical significance as ns, no significant difference, *p < 0.05, **p < 0.01, ***p < 0.001, and ****p < 0.0001.

Natural aging is a gradual physiological process. To assess ARHL, an aging model was generated by treating HEI‐OC1 cells with various concentrations of D‐gal (2, 5, 10, 20, and 40 mg/mL) for 72 h. CCK‐8 assay showed that with an increase in D‐gal concentration, HEI‐OC1 cell viability gradually reduced (Figure 1A,B). Based on these results, HEI‐OC1 cells were treated with low (5 mg/mL) and high (20 mg/mL) concentrations of D‐gal to determine the levels of reactive oxygen species (ROS). Immunofluorescence staining and flow cytometry with MitoSOX and DCFH‐DA indicated increased levels of ROS in D‐gal‐treated HEI‐OC1 cells after 72 h (Figure 1C–J). Subsequently, aging‐related marker expression levels of SMP30, Lamin B1, γ‐H2A.X, P21, and P16 were detected in HEI‐OC1 cells exposed to different D‐gal concentrations (Matias et al. 2022; Shao et al. 2021; Lopez‐Otin et al. 2023). SMP30 and Lamin B1 were downregulated in HEI‐OC1 cells exposed to high concentrations of D‐gal. However, the expression levels of SMP30 and Lamin B1 increased abnormally after treatment with low D‐gal concentrations (Figure 1K,L,N,O, Figure S1A,B,D,E), indicating that low D‐gal levels may activate cellular self‐protection mechanisms. With the increasing concentration of D‐gal, the expression of γ‐H2A.X, P21, and P16 was increased gradually (Figure 1M,P, Figure S1C,F,G–I,K). Immunofluorescence staining revealed that the number of γ‐H2A.X aggregation spots in D‐gal‐treated HEI‐OC1 cells increased significantly (Figure S1J,L), consistent with the Western blotting results. Additionally, the SA‐β‐gal staining results revealed that cellular aging was obviously elevated in HEI‐OC1 cells treated with D‐gal (Figure S1M,N). These results indicate that treatment with 20 mg/mL D‐gal for 72 h effectively induces cellular aging in HEI‐OC1 cells, providing a robust model for studying aging.

**FIGURE 2:**
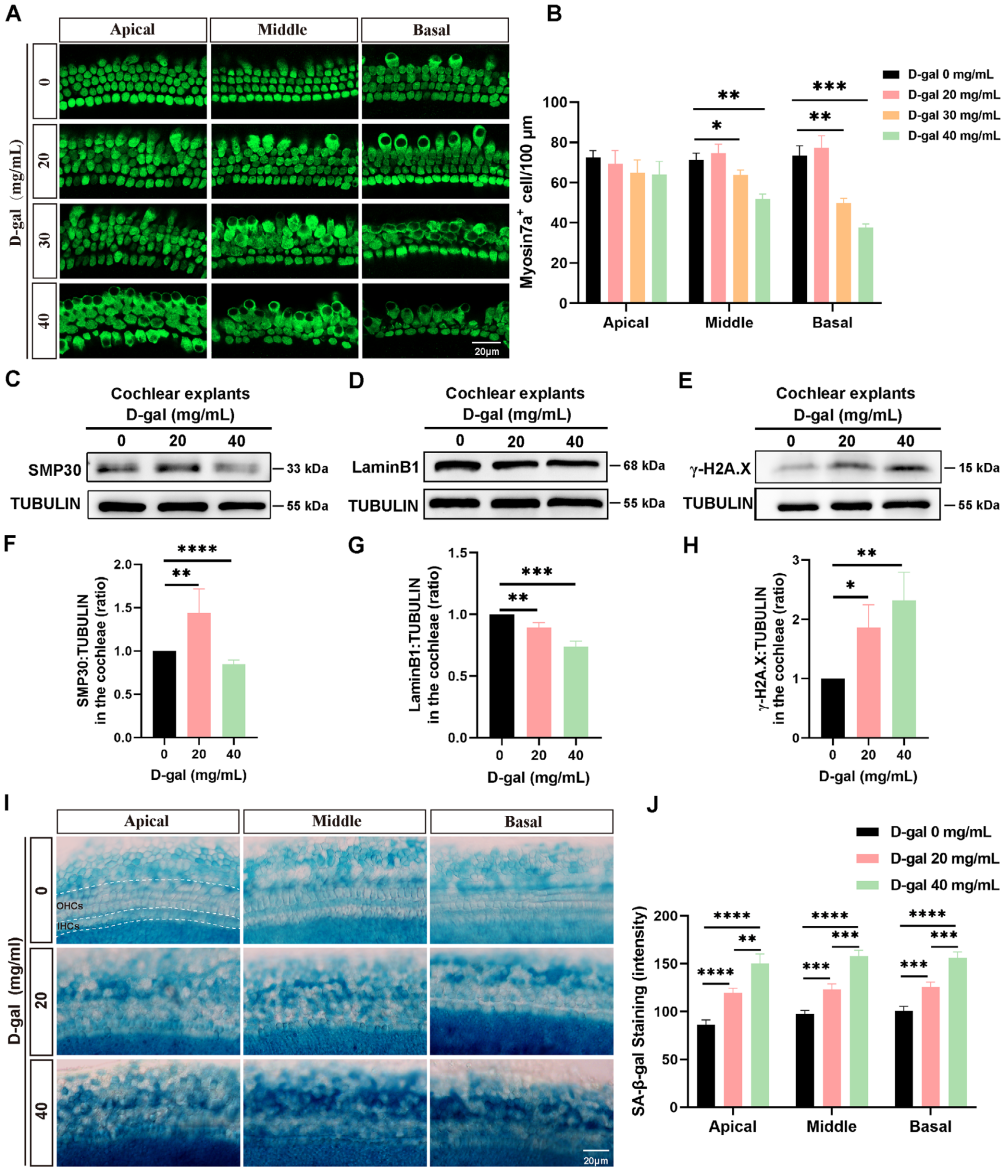
Establishment of D‐gal‐induced aging model in cochlear explants. (A) Immunofluorescence staining demonstrating the number of myosin7a^+^ hair cells in cochlear explants treated with 20, 30, and 40 mg/mL D‐gal for 72 h. (B) Quantification of the number of myosin7a^+^ hair cells in cochlear explants (*n* = 4; four individual explants from four mice). (C–E) Western blotting of senescence marker protein‐30 (SMP30), Lamin B1, and γ‐H2A.X expression in cochlear explants exposed to D‐gal. (F–H) Statistical analysis of SMP30 (*n* = 5), Lamin B1 (*n* = 3), and γ‐H2A.X (*n* = 3) in C–E; *n* corresponds to the number of independent samples, each consisting of 12 explants from six mice. (I) Senescence‐associated β‐gal staining images of hair cells in cochlear explants exposed to D‐gal. (J) Quantification of β‐gal staining intensity in I (*n* = 4; four individual explants from four mice). Scale bar: 20 μm. Statistical significance is indicated as *p < 0.05, **p < 0.01, ***p < 0.001, and ****p < 0.0001.

To establish a cochlear explant aging model, the cochlear basilar membrane was dissected from P3 mice and cultured in vitro. Based on a previous report (Guo et al. 2020), cochlear explants were exposed to various concentrations of D‐gal for 72 h. Cochlear explants were subjected to immunofluorescence staining for myosin7a—a marker of hair cells, revealing that no significant change in the number of myosin7a^+^ cells was observed in the low D‐gal concentration (20 mg/mL) group, indicating that low concentration (20 mg/mL) did not cause the loss of cochlear hair cells. However, the number of myosin7a^+^ cells in the high‐concentration D‐gal treatment group did not alter significantly in the apical region but reduced significantly in the middle and basal regions (Figure 2A,B), indicating that high concentration of D‐gal (40 mg/mL) induced an aging phenotype and caused cochlear hair cell damage. SMP30 was downregulated in cochlear explants exposed to high concentration of D‐gal (40 mg/mL), whereas it was abnormally upregulated after treatment with low D‐gal concentration (Figure 2C,F, Figure S2A,D). With an increase in D‐gal concentration, the expression level of Lamin B1 was reduced (Figure 2D,G, Figure S2B,E), whereas that of γ‐H2A.X, P21, and P16 were increased significantly (Figure 2E,H, Figure S2C,F,G–J). Additionally, SA‐β‐gal staining intensity was significantly upregulated in cochlear explants exposed to D‐gal (Figure 2I,J). These findings indicated that exposure to 40 mg/mL D‐gal for 72 h effectively induces aging in cochlear hair cells in vitro. D‐gal‐treated HEI‐OC1 cell and cochlear explant aging models offer a valuable framework for assessing the mechanisms of aging cochlear hair cell damage and developing potential therapeutic approaches for ARHL.

### 3.2 NMNAT1 Expression Levels Were Reduced in Aging HEI‐OC1 Cells and Cochlear Explants Treated With D‐Gal

**FIGURE 3:**
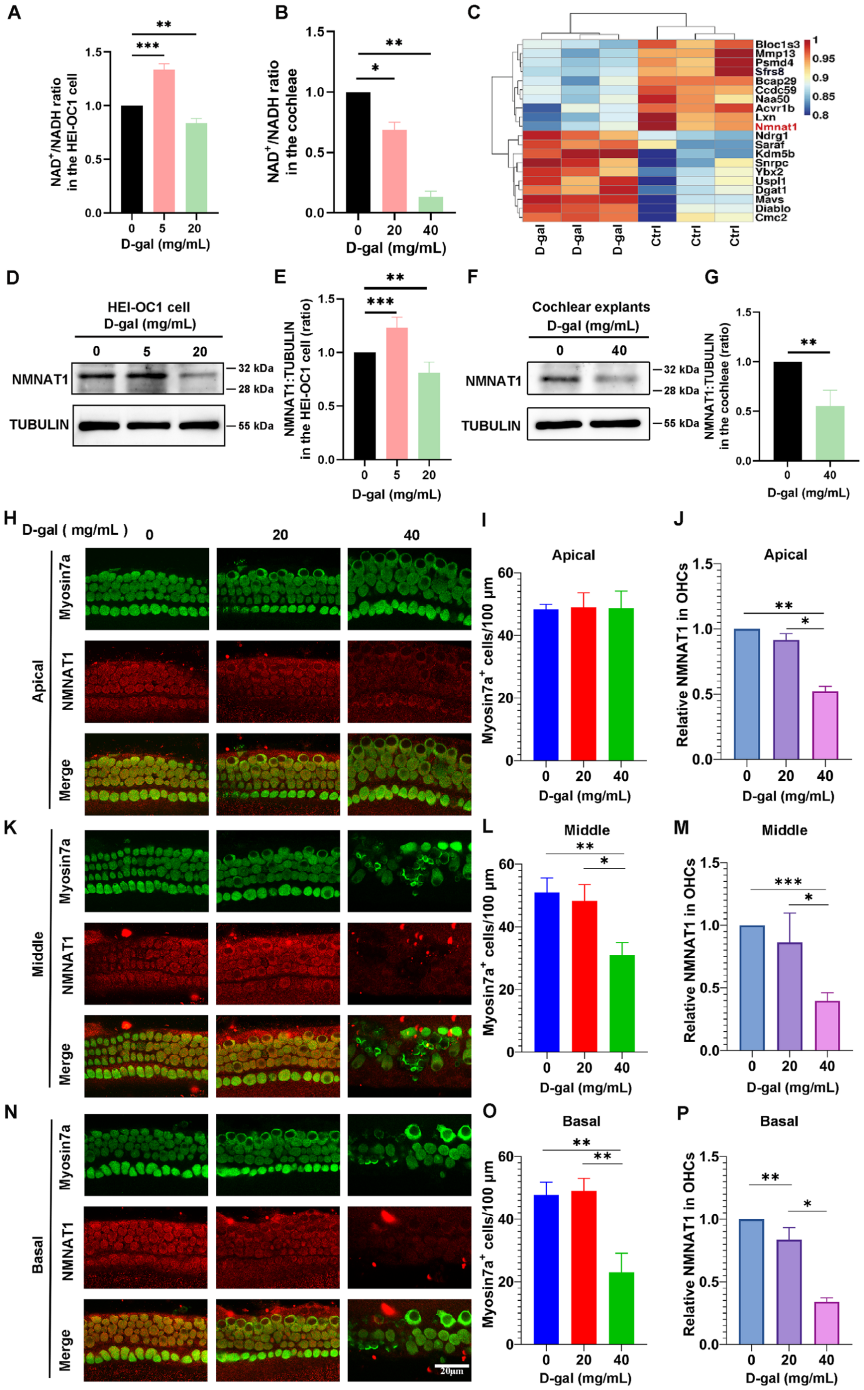
NMNAT1 levels are decreased in D‐gal‐treated aging of HEI‐OC1 cells and cochlear explants. (A) NAD^+^ levels are significantly reduced in D‐gal treated HEI‐OC1 cells for 72 h (*n* = 3, independent samples). (B) NAD^+^ levels are significantly reduced in the D‐gal‐induced cochlear explant aging model (*n* = 4, independent samples, each consisting of 12 explants from six mice). (C) Results of proteomic analysis demonstrating the significant downregulation of NMNAT1 in the D‐gal‐induced aging model (*n* = 3, independent samples). (D) Western blotting of NMNAT1 expression in HEI‐OC1 cells exposed to D‐gal (5 and 20 mg/mL). (E) Statistical analysis of NMNAT1 expression in D (*n* = 5, independent samples). (F) Western blotting of NMNAT1 expression in cochlear explants exposed to D‐gal (40 mg/mL) for 72 h. (G) Quantification of NMNAT1 expression in F (*n* = 3, independent samples, each consisting of 12 explants from six mice). (H) Immunofluorescence staining demonstrating the number of myosin7a^+^ hair cells and NMNAT1 levels in 20 and 40 mg/mL D‐gal‐treated apical cochlear explants. (I) Statistical analysis demonstrating the number of myosin7a^+^ hair cells in the apical cochlear explants (*n* = 4; four individual explants from four mice). (J) Statistical analysis demonstrating NMNAT1 levels in outer hair cells (OHCs) in the apical cochlear explants (*n* = 4; four individual explants from four mice). (K) Immunofluorescence staining demonstrating the number of myosin7a^+^ hair cells and NMNAT1 levels in the middle cochlear explants. (L) Quantification of the number of myosin7a^+^ hair cells in the middle cochlear explants (*n* = 4; four individual explants from four mice). (M) Statistical analysis of NMNAT1 levels in OHCs in the middle cochlear explants (*n* = 4; four individual explants from four mice). (N) Immunofluorescence staining demonstrating the number of myosin7a^+^ hair cells and NMNAT1 levels in the basal cochlear explants. (O) Statistical analysis demonstrating the number of myosin7a^+^ hair cells in the basal cochlear explants (*n* = 4; four individual explants from four mice). (P) Statistical analysis demonstrating NMNAT1 levels in OHCs in the basal cochlear explants (*n* = 4; four individual explants from four mice). Scale bar: 20 μm. Statistical significance is indicated as *p < 0.05, **p < 0.01, and ***p < 0.001.

NAD^+^ levels are obviously decreased in the cochlear cells of aged mice (Okur et al. 2023). Additionally, we identified that NAD^+^ levels in HEI‐OC1 cells exposed to D‐gal (20 mg/mL) were significantly reduced (Figure 3A). Moreover, NAD^+^ levels were significantly reduced with an increase in D‐gal concentrations in the D‐gal‐treated cochlear explant aging model (Figure 3B). To assess alterations in gene expression in D‐gal‐induced aging cells, we used proteomics to detect protein expression in HEI‐OC1 cells exposed to 20 mg/mL D‐gal. The proteomic results revealed that NMNAT1 expression was significantly reduced in the D‐gal‐induced aging model (Figure 3C).

NMNAT1 is a core enzyme involved in NAD^+^ biosynthesis and is essential for maintaining cellular NAD^+^ levels. Alterations in NAD^+^ levels are associated with a reduced NMNAT1 expression. Human *Nmnat1* is predominantly expressed in the nucleus (Lau et al. 2010); however, its localization in mice remains unclear. To determine the subcellular localization of NMNAT1 in mouse‐derived HEI‐OC1 cells, we performed a nucleoplasmic separation assay and observed that NMNAT1 was expressed in both the cytoplasm and nucleus, with a predominant localization in the nucleus (Figure S3A,B). Additionally, immunofluorescence staining revealed that NMNAT1 was expressed in the nucleus and cytoplasm of mouse cochlear hair cells (Figure S3C), consistent with the results of the nucleoplasmic separation assay.

To verify the proteomics results, we performed western blot and observed that NMNAT1 was slightly upregulated in HEI‐OC1 cells exposed to low concentration of D‐gal (5 mg/mL), but significantly downregulated at high concentration of D‐gal (20 mg/mL) (Figure 3D,E). Alterations in NMNAT1 expression were further confirmed in D‐gal‐exposed aging cochlear hair cells, with significantly reduced NMNAT1 levels in the high‐concentration D‐gal (40 mg/mL) treatment group (Figure 3F,G). These results indicate that NMNAT1 levels were significantly reduced in D‐gal‐treated aging HEI‐OC1 and cochlear hair cells.

To further confirm these findings, immunofluorescence staining revealed that NMNAT1 expression levels in cochlear OHCs in the apical, middle, and basal regions were significantly reduced in the high‐concentration D‐gal (40 mg/mL) group (Figure 3H,J,K,M,N,P). Specifically, the expression level of NMNAT1 in cochlear hair cells in the basal region gradually reduced with increasing D‐gal concentrations (Figure 3N,P), indicating that cochlear basal hair cells were more sensitive to D‐gal induction and more susceptible to damage. These results indicated that D‐gal induces cochlear hair cell damage and alters NMNAT1 expression in aging hair cells, supporting the potential role of NMNAT1 in aging hair cells.

### 3.3 Autophagy Was Impaired in the D‐Gal‐Induced Aging Model, and *Nmnat1* Overexpression Activated Autophagy and Delayed Aging

**FIGURE 4:**
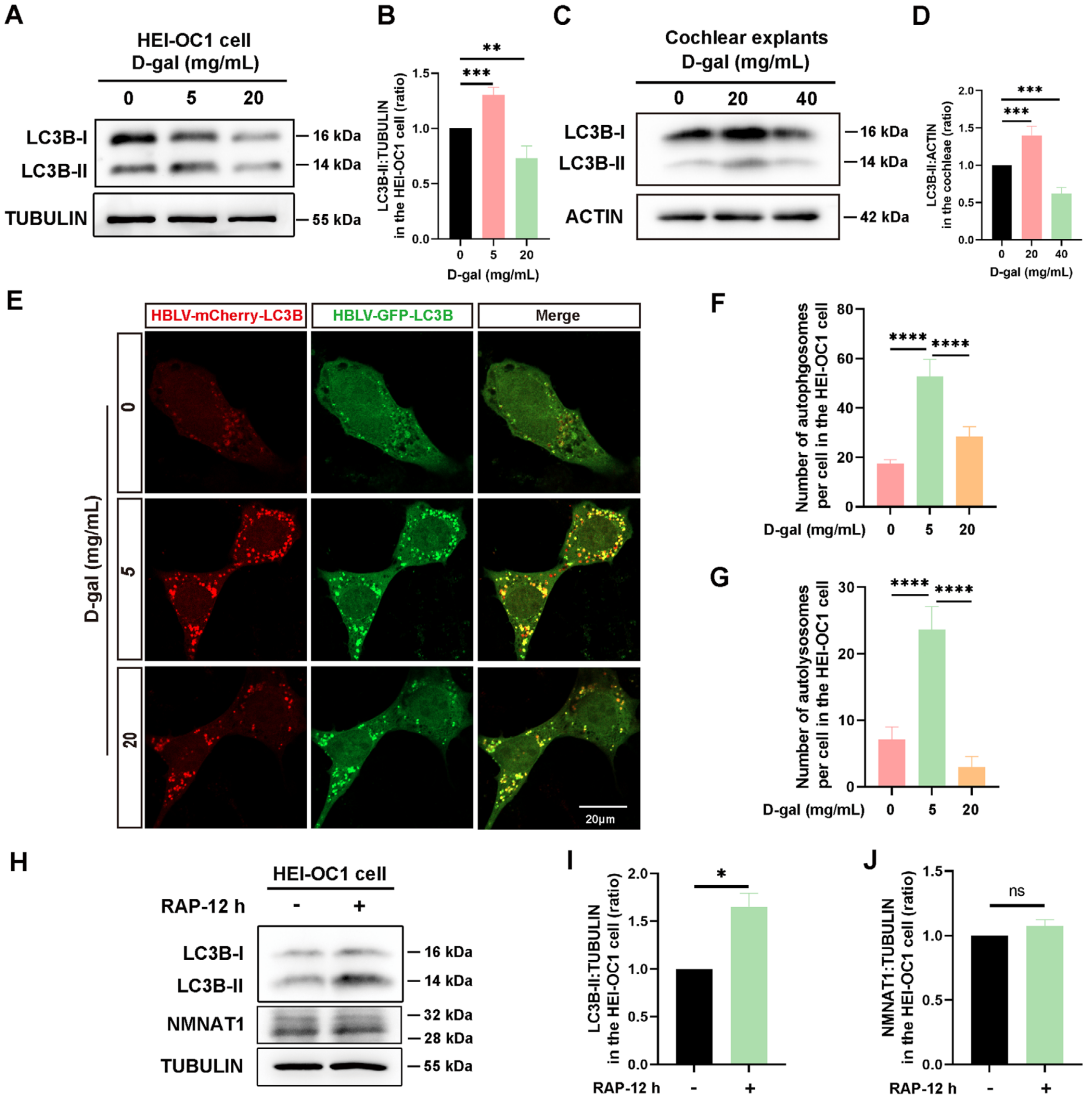
D‐gal‐induced aging impairs autophagy in HEI‐OC1 cells and cochlear explants. (A) Western blotting of LC3B‐II expression in D‐gal treated HEI‐OC1 cells. (B) Statistical analysis of LC3B‐II expression in A (*n* = 4, independent samples). (C) Western blotting of LC3B‐II expression in D‐gal treated cochlear explants. (D) Quantitative analysis of LC3B‐II expression in C (*n* = 4, independent samples, each consisting of 12 explants from six mice). (E) Autophagic flux is assessed by monitoring mCherry‐GFP‐LC3B expression in D‐gal treated HEI‐OC1 cells. Immunofluorescence staining demonstrating the number of yellow (autophagosomes) and red puncta (autolysosomes). (F) Statistical analysis of the number of autophagosomes (yellow puncta) in E (*n* = 10; 10 cells from four independent samples). (G) Quantitative analysis of the number of autolysosomes (red puncta) in E (*n* = 10; 10 cells from four independent samples). (H) Western blotting of LC3B‐II and nicotinamide nucleotide adenylate transferase 1 (NMNAT1) expression in HEI‐OC1 cells exposed to 100 nM rapamycin for 12 h. (I) Quantification of LC3B‐II expression in H (*n* = 3, independent samples). (J) Statistical analysis of NMNAT1 expression in H (*n* = 3, independent samples). Scale bar: 20 μm. Statistical significance is indicated as *p < 0.05, **p < 0.01, ***p < 0.001, ****p < 0.0001, and ns, no significant difference.

Autophagy levels gradually decline with age, and there is an association between autophagy dysfunction and aging (Aman et al. 2021; Kaushik et al. 2021; Wong et al. 2020). To assess whether autophagy levels were altered in the D‐gal‐induced aging model, we determined that LC3B‐II, ATG7, and BECLIN1 expression levels were increased after treatment with low D‐gal concentration (5 mg/mL) but were obviously reduced after exposure to high D‐gal concentration (20 mg/mL) in HEI‐OC1 cells (Figure 4A,B, Figure S4A,C,D,F,G,J). P62 expression levels gradually increased after exposure to D‐gal for 72 h (Figure S4B,E). Additionally, the expression levels of LC3B‐II, P62, ATG7, and BECLIN1 in D‐gal‐induced aging cochlear explants aligned with the results obtained from D‐gal‐treated aging of HEI‐OC1 cells (Figure 4C,D, Figure S4H,I,K–P). These results indicated that low D‐gal concentrations activate autophagy, whereas high concentrations suppress it.

To assess alterations in autophagy levels, HEI‐OC1 cells were infected with HBLV‐mCherry‐GFP‐LC3B lentivirus to establish a stable cell line. Western blot results confirmed that the expression of endogenous LC3B was not affected by exogenous mCherry‐GFP‐LC3B in the stable cell line (Figure S5F). Following D‐gal‐induced aging, autophagic flux was assessed by monitoring mCherry‐GFP‐LC3B expression. The number of yellow (autophagosomes) and red puncta (autolysosomes) significantly increased in cells treated with a low D‐gal concentration (5 mg/mL). In contrast, treatment with a high D‐gal concentration (20 mg/mL) significantly reduced both types of puncta compared with the low concentration group (Figure 4E–G). These results align with our previous findings (Wei et al. 2025), indicating that low D‐gal concentration activates autophagy, whereas high D‐gal concentration significantly impairs autophagic activity in aging HEI‐OC1 cells. These results indicate that autophagy was impaired in D‐gal‐induced aging of HEI‐OC1 cells and cochlear explants.

*hNmnat1* Overexpression in AD facilitates autophagy and reduces amyloid plaques (Zhu et al. 2022). NMNAT1 activates autophagy by regulating the SIRT1/mTOR pathway in aged rats (Wang et al. 2019). To assess the relationship between NMNAT1 and autophagy in the D‐gal‐treated aging model, HEI‐OC1 cells were treated with 100 nM rapamycin for 12 h. Western blotting revealed a significant increase in LC3B‐II expression (Figure 3H,I). However, NMNAT1 expression was unaffected (Figure 4H–J). Similar results were obtained after rapamycin treatment for 24 h (Figure S5A–C). These results indicated that NMNAT1 expression was not affected by autophagic activation.

**FIGURE 5:**
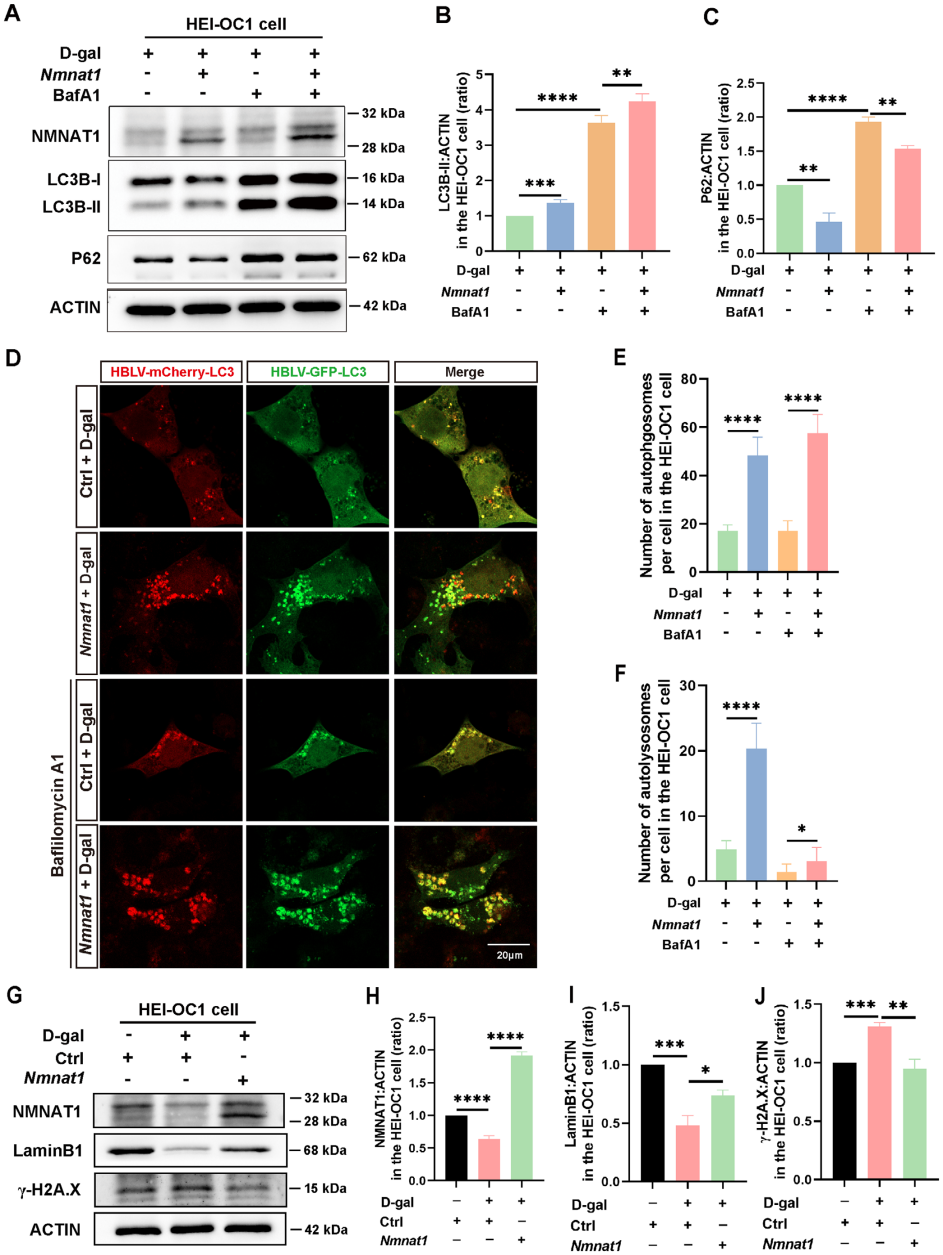
Overexpression of *Nmnat1* activates autophagy and delays aging in D‐gal‐treated HEI‐OC1 cells. (A) Western blot results of microtubule‐associated protein 1 light chain 3 beta (LC3B‐II) and P62 in *Nmnat1*‐overexpressing HEI‐OC1 cells exposed to D‐gal (20 mg/mL) for 72 h. The cells are exposed to 100 nM bafilomycin A1 for 12 h before collection. (B) Statistical analysis of LC3B‐II expression in A (*n* = 4, independent samples). (C) Statistical analysis of P62 expression in A (*n* = 3, independent samples). (D) Immunofluorescence staining demonstrating that *Nmnat1* overexpression significantly increased the number of yellow and red puncta in D‐gal‐treated aging HEI‐OC1 cells. (E) Statistical analysis of the number of autophagosomes (yellow puncta) in D (*n* = 14; 14 cells from four independent samples). (F) Statistical analysis of the number of autolysosomes (red puncta) in D (*n* = 14; 14 cells from four independent samples). (G) Western blotting of Lamin B1 and γ‐H2A.X expression in NMNAT1‐overexpressing HEI‐OC1 cells. (H) Statistical analysis of NMNAT1 expression in G (*n* = 3, independent samples). (I) Statistical analysis of lamin B1 expression in G (*n* = 3, independent samples). (J) Statistical analysis of γ‐H2A.X expression in G (*n* = 3, independent samples). Scale bar: 20 μm. Statistical significance is indicated as *p < 0.05, **p < 0.01, ***p < 0.001, and ****p < 0.0001.

To confirm the role of NMNAT1 in autophagy regulation, NMNAT1 was overexpressed in HEI‐OC1 cells by transfection with the pAAV‐CMV‐*Nmnat1*‐EGFP plasmid for 12 h, followed by treatment with D‐gal (20 mg/mL) for 72 h. Subsequently, HEI‐OC1 cells were treated with bafilomycin A1 (100 nM) for 12 h before cell collection. Notably, western blotting revealed that *Nmnat1* overexpression upregulated LC3B‐II (Figure 5A,B) and downregulated P62 (Figure 5A,C). However, BECLIN1 levels remained unaltered (Figure S5D,E). Compared with bafilomycin A1 treatment alone, combined treatment with *Nmnat1* overexpression and bafilomycin A1 further increased LC3B‐II levels (Figure 5A,B) and slightly reduced P62 levels (Figure 5A,C). To further affirm these findings, autophagic flux was evaluated using mCherry‐GFP‐LC3B expression. *Nmnat1* overexpression increased the number of yellow (autophagosomes) and red puncta (autolysosomes) in D‐gal‐induced aging HEI‐OC1 cells, and co‐treatment with bafilomycin A1 further enhanced their accumulation (Figure 5D–F). These findings indicated that *Nmnat1* overexpression enhances autophagic flux and may modulate the aging process by regulating autophagy in the D‐gal‐induced aging model.

Autophagy activation in aging cochlear hair cells facilitates their survival (He, Li, et al. 2021). To confirm the effect of NMNAT1 on aging, HEI‐OC1 cells were transfected with the *Nmnat1* plasmid for 12 h and then treated with D‐gal (20 mg/mL) for 72 h. Western blotting revealed that Lamin B1 was significantly upregulated in the NMNAT1 overexpression group compared to the D‐gal‐treated group alone (Figure 5G–I), whereas the expression level of γ‐H2A.X was significantly reduced (Figure 5G,J). These results demonstrate that *Nmnat1* overexpression can delay D‐gal‐induced aging by activating autophagy.

### 3.4 Overexpression of *Nmnat1* Increased Protected Cochlear Hair Cells From D‐Gal‐Induced Aging in Cochlear Explants In Vitro

**FIGURE 6:**
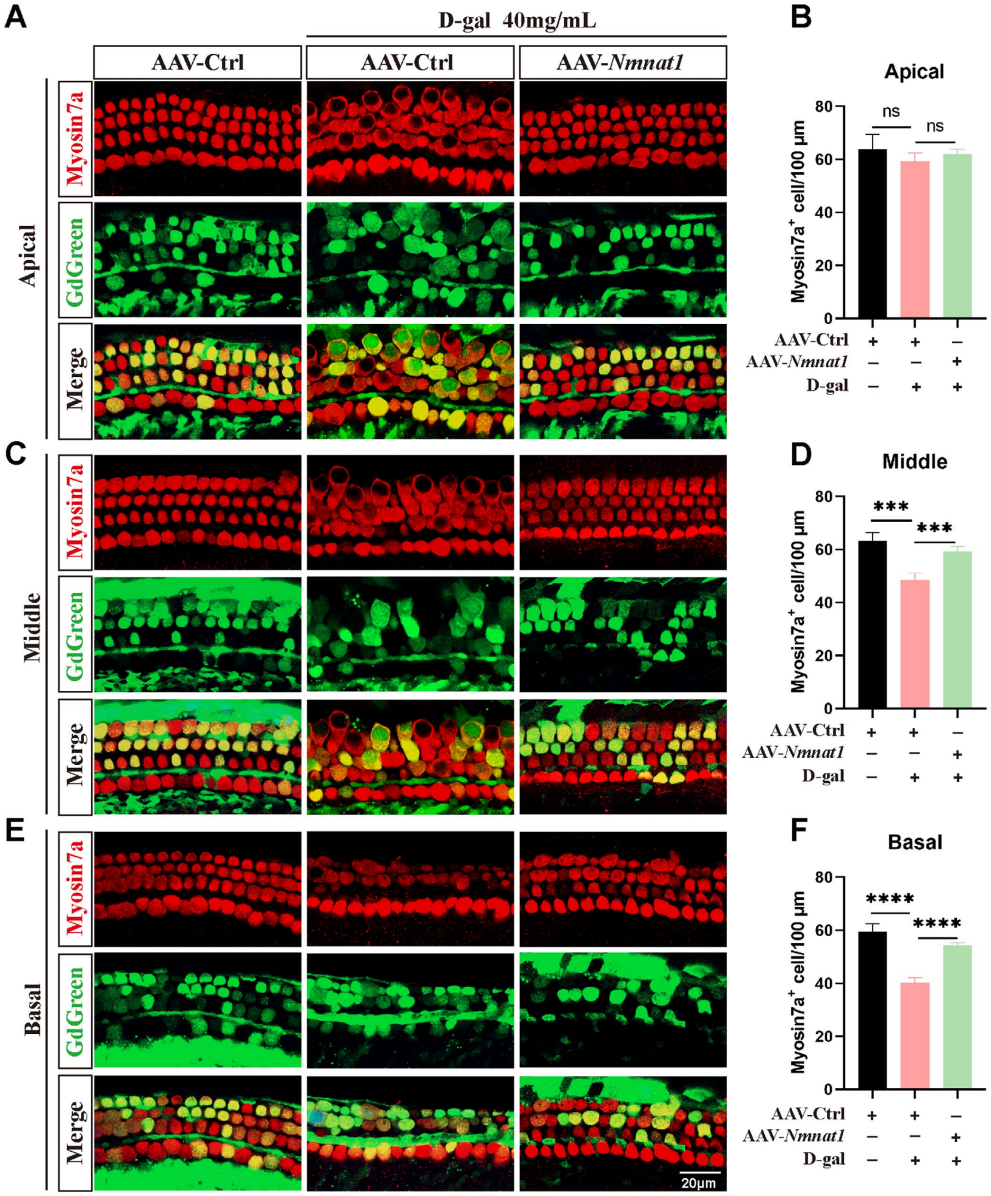
Overexpression of *Nmnat1* by AAV2.7m8‐*Nmnat1* attenuated D‐gal–induced damage and enhanced hair cell survival. (A) Immunofluorescence staining demonstrating the number of myosin7a^+^ hair cells in AAV2.7m8‐*Nmnat1*‐infected and 40 mg/mL D‐gal‐treated apical cochlear explants. (B) Statistical analysis of the number of myosin7a^+^ hair cells in the apical cochlear explants (*n* = 4; four individual explants from four mice). (C) Immunofluorescence staining demonstrating the number of myosin7a^+^ hair cells in the middle cochlear explants. (D) Statistical analysis of myosin7a^+^ hair cells in the middle cochlear explants (*n* = 4; four individual explants from four mice). (E) Immunofluorescence staining demonstrating myosin7a^+^ hair cells in the basal cochlear explants. (F) Statistical analysis of myosin7a^+^ hair cells in the basal cochlear explants (*n* = 4; four individual explants from four mice). Scale bar: 20 μm. Statistical significance is indicated as ***p < 0.001, ****p < 0.0001, and ns, no significant difference.

To verify the role of NMNAT1 in cochlear hair cells, we constructed AAV2.7m8‐*Nmnat1* virus. AAV2.7m8 is highly efficient in infecting cochlear hair cells (Isgrig et al. 2019). Cultured cochlear explants were infected with AAV2.7m8‐*Nmnat1* in vitro for 36 h, followed by treatment with 40 mg/mL D‐gal for 72 h. Immunofluorescence staining revealed that the number of myosin7a^+^ hair cells was obviously decreased in the middle and basal turns of the D‐gal‐alone treatment group (Figure 6A–F). However, the number of myosin7a^+^ hair cells was higher in the middle and basal turns of the AAV2.7m8‐*Nmnat1* and D‐gal treatment groups than that in the D‐gal‐alone treatment group (Figure 6A–F). These results indicate that *Nmnat1* overexpression attenuates D‐gal‐treated cochlear hair cell damage and increases cochlear hair cell survival.

### 3.5 Tricarboxylic Acid Cycle (TCA) Was Impaired in *Nmnat1*‐KO Cells

NAD^+^ is a primary coenzyme in oxidation–reduction reactions, whereas NADH is the reduced form that is essential for maintaining energy metabolism and regulating various dehydrogenases involved in metabolic pathways (Verdin 2015; Ross 2021). To assess the biological function of *Nmnat1* in HEI‐OC1 cells, we constructed an *Nmnat1*‐KO cell line using CRISPR/Cas9 technology in HEI‐OC1 cells. RT‐qPCR and western blot analyses indicated that *Nmnat1* expression was successfully reduced at both the mRNA and protein levels in the *Nmnat1*‐KO cells (Figure S6A–C). To predict the potential off‐target sites of CRISPR/Cas9 technology, we predicted the off‐target sites of sgRNAs using the CCTop‐CRISRP/Cas9 target online predictor website (Figure S6D,E). Analysis of potential off‐target sites confirmed the absence of unintended editing by sgRNA1 and sgRNA2 in Nmnat1‐KO cell lines (Figure S6F–H). These results indicate that *Nmnat1* was effectively knocked out without off‐target effects.

**FIGURE 7:**
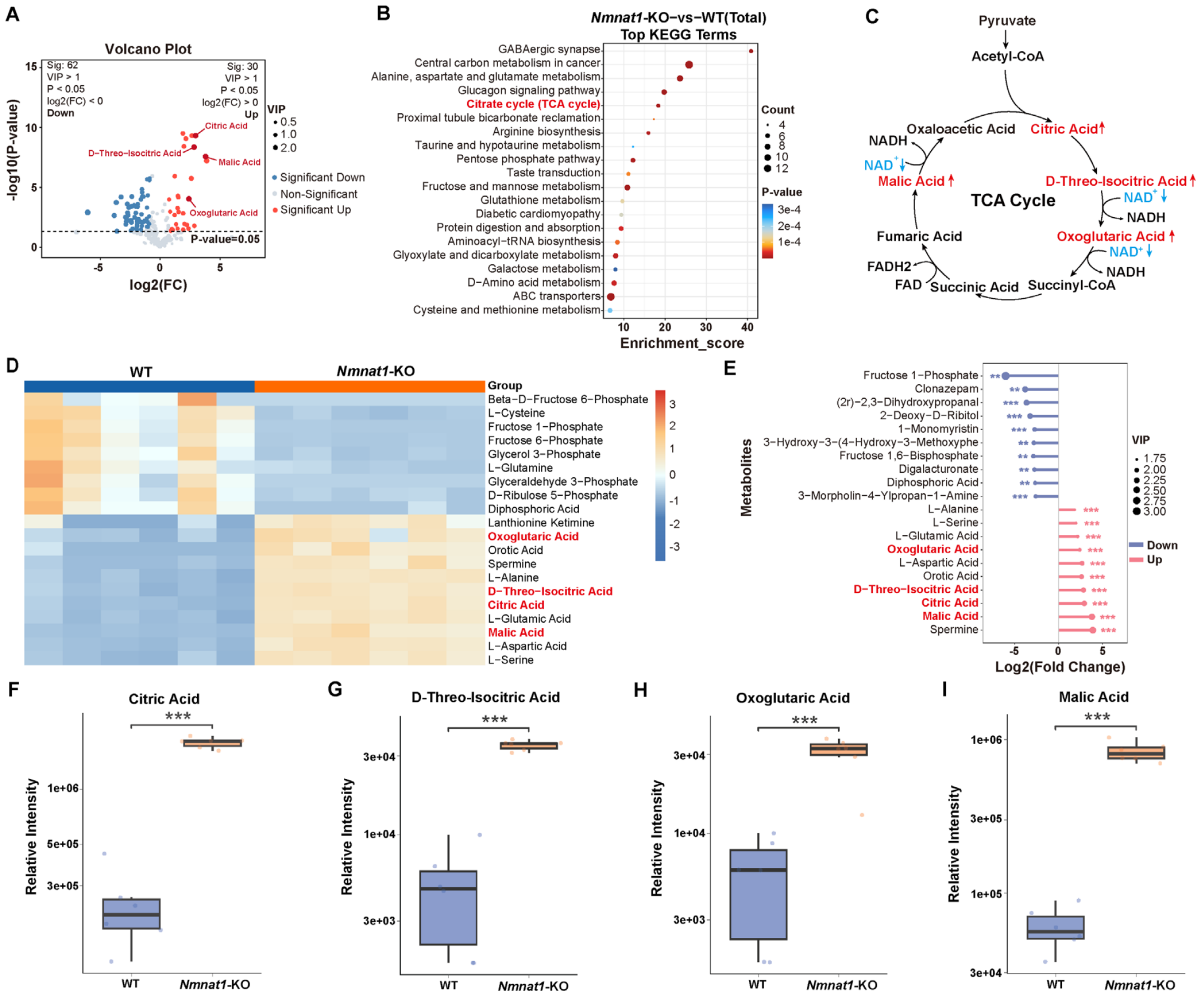
Metabolomic analysis demonstrating impaired tricarboxylic acid cycle (TCA) metabolism in *Nmnat1‐KO* cells. (A) Volcano plots demonstrating the differential expression of various metabolites in *Nmnat1*‐KO cells. (B) Bubble map of Kyoto Encyclopedia of Genes and Genomes enrichment analysis of signaling pathways associated with metabolism. (C) Schematic illustration of the TCA metabolism. (D) Heatmap demonstrating the differential expression of various metabolites in *Nmnat1*‐KO cells. (E) Lollipop map demonstrating the metabolites and their log2 (Fold Change) values. (F–I) Boxplot demonstrating the relative expression levels of citric acid, D‐threo‐isocitric acid, oxoglutaric acid, and malic acid in *Nmnat1*‐KO cells (*n* = 6, independent samples). Statistical significance is indicated as **p < 0.01 and ***p < 0.001.

To assess the effects of NMNAT1 on cellular metabolic pathways, we determined the differential metabolite profiles of HEI‐OC1 and *Nmnat1*‐KO cells using untargeted metabolomics. Volcano plots revealed the differential expression of various metabolites in *Nmnat1*‐KO cells (Figure 7A). Pathway enrichment analysis revealed that multiple metabolic pathways were affected in *Nmnat1*‐KO cells, including amino acid and glucose metabolism (Figure 7B). Notably, the TCA cycle was disrupted in *Nmnat1*‐KO cells, with an abnormal accumulation of multiple TCA cycle intermediates, including citric acid, D‐threo‐isocitric acid, oxoglutaric acid, and malic acid (Figure 7B–E). The TCA cycle is the hub of intracellular material and energy metabolism (Martinez‐Reyes and Chandel 2020). The conversion of citric acid and D‐threo‐isocitric acid to oxoglutaric acid, oxoglutaric acid to succinyl coenzyme A, and malic acid to oxaloacetic acid requires NAD^+^ (Figure 7C). NMNAT1 is the primary rate‐limiting enzyme in NAD^+^ biosynthesis, and its deletion blocks NAD^+^ synthesis, disrupting the TCA cycle and causing the accumulation of citric acid, D‐threo‐isocitric acid, oxoglutaric acid, and malic acid owing to impaired subsequent metabolism (Figure 7F–I). These results revealed a significant regulatory role of NMNAT1 in cellular metabolism.

## 4 Discussion

Current research on ARHL is limited because of its complex etiology. D‐gal is a reducing sugar that is typically metabolized to glucose at physiological concentrations. However, its accumulation at high concentrations disrupts cellular metabolism and increases cellular osmolality and ROS production, thereby facilitating aging. This accumulation induces pathological alterations that closely resemble those of natural aging (Guo et al. 2020; Aman et al. 2021; Saleh et al. 2019). Therefore, D‐gal is commonly used to establish aging models.

In this study, we observed that the levels of aging markers SMP30 (Figure 1K,N, Figure S1A,D) and Lamin B1 (Figure 1L,O, Figure S1B,E) were abnormally increased in HEI‐OC1 cells exposed to low (5 mg/mL) D‐gal concentrations, along with an increase in NAD^+^ levels (Figure 3A) and autophagy (Figure 4A, Figure S4A,D). These alterations differed from typical aging cell profiles, indicating that low‐concentration D‐gal treatment may activate a cellular self‐protection mechanism in HEI‐OC1 cells, thereby increasing autophagy levels. Our findings align with the report by He, Li, et al. (2021), which reported enhanced autophagy in HEI‐OC1 cells exposed to low concentration of D‐gal. Additionally, we observed that NMNAT1 was upregulated in the D‐gal‐treated aging model at low D‐gal concentrations, whereas it was significantly downregulated at high D‐gal concentrations (Figure 3D,E). This aligns with the alterations in NAD^+^ and autophagy levels, indicating that low D‐gal concentrations may trigger self‐protective responses in HEI‐OC1 cells by activating NMNAT1 and associated signaling pathways to facilitate cell survival. In contrast, a high D‐gal concentration diminished these protective mechanisms.

The NMNAT family of proteins—that forms a primary enzyme group catalyzing NAD^+^ biosynthesis—comprises three primary isoforms distinguished by their subcellular localization: NMNAT1, NMNAT2, and NMNAT3 localized in the nucleus, Golgi, and mitochondria in humans, respectively (Lau et al. 2010; Jayaram et al. 2011). hNMNAT1 localizes to the nucleus of human cells, as confirmed by the expression of recombinant proteins in *Escherichia coli* (Schweiger et al. 2001). However, NMNAT1 localization in mouse cells remains unclear. In this study, immunofluorescence staining of NMNAT1 in cochlear hair cells demonstrated predominant nuclear localization, with additional cytoplasmic expression (Figure S3C). To confirm NMNAT1 localization in HEI‐OC1 cells, we determined its expression in the nuclear and cytoplasmic fractions. Although NMNAT1 was detected in both fractions, its levels were higher in the nuclear fraction (Figure S3A,B). Additionally, cytoplasmic *Nmnat1* overexpression in retinal ganglion cells and axons of transgenic mice enhanced the survival of these cells (Zhu et al. 2013). These findings indicate that although NMNAT1 is predominantly nuclear, its cytoplasmic expression may play a significant role, requiring further assessment.

NAD^+^ levels decline with age. As a central metabolic cofactor, NAD^+^ regulates numerous metabolic pathways and fluxes (Covarrubias et al. 2021; Verdin 2015). Therefore, maintaining NAD^+^ homeostasis is essential for preventing age‐related metabolic dysfunctions. Interventions that modulate NAD^+^ levels or supplement NAD^+^ precursors have demonstrated promise in alleviating metabolic disorders, including abnormalities in glucose and lipid metabolism (Tarrago et al. 2018; Yoshino et al. 2011). In this study, NMNAT1 deletion disrupted the TCA cycle (Figure 7B–I) and significantly reduced glucose, fructose, mannose, and galactose metabolism (Figure 7B,D). This likely resulted from impaired NAD^+^ synthesis because of NMNAT1 deficiency, indicating the crucial regulatory role of NMNAT1 in cellular metabolism. Although NMNAT1 expression is widely reported to regulate cellular NAD^+^ levels, a study on AD revealed that NMNAT1 overexpression enhances NMNAT enzymatic activity without altering NAD^+^ levels (Rossi et al. 2018). A negative feedback mechanism has been hypothesized to maintain NAD^+^ homeostasis under these conditions. Recent studies have increasingly highlighted the association between autophagy dysfunction and aging. Autophagy and cellular metabolic functions decline with age (Wong et al. 2020). Autophagy initiation and activation are energy‐intensive processes that are affected by nutrient availability and external signals. Dysregulation of glucose and cholesterol affects the mTOR and AMP‐activated protein kinase signaling pathways, impairing autophagy, and promoting aging (Castellano et al. 2017; Wolfson et al. 2017; Zhou et al. 2017). In contrast, autophagy sustains cellular metabolic homeostasis under nutrient‐limited conditions by providing precursors for anabolism and energy metabolism. During starvation, the autophagic breakdown of adipose tissue and muscle helps maintain blood glucose levels and provides glucose and ketone bodies to support neuronal metabolism in the brain (Khawar et al. 2019; Lin et al. 2021; Yang et al. 2019).

**FIGURE 8:**
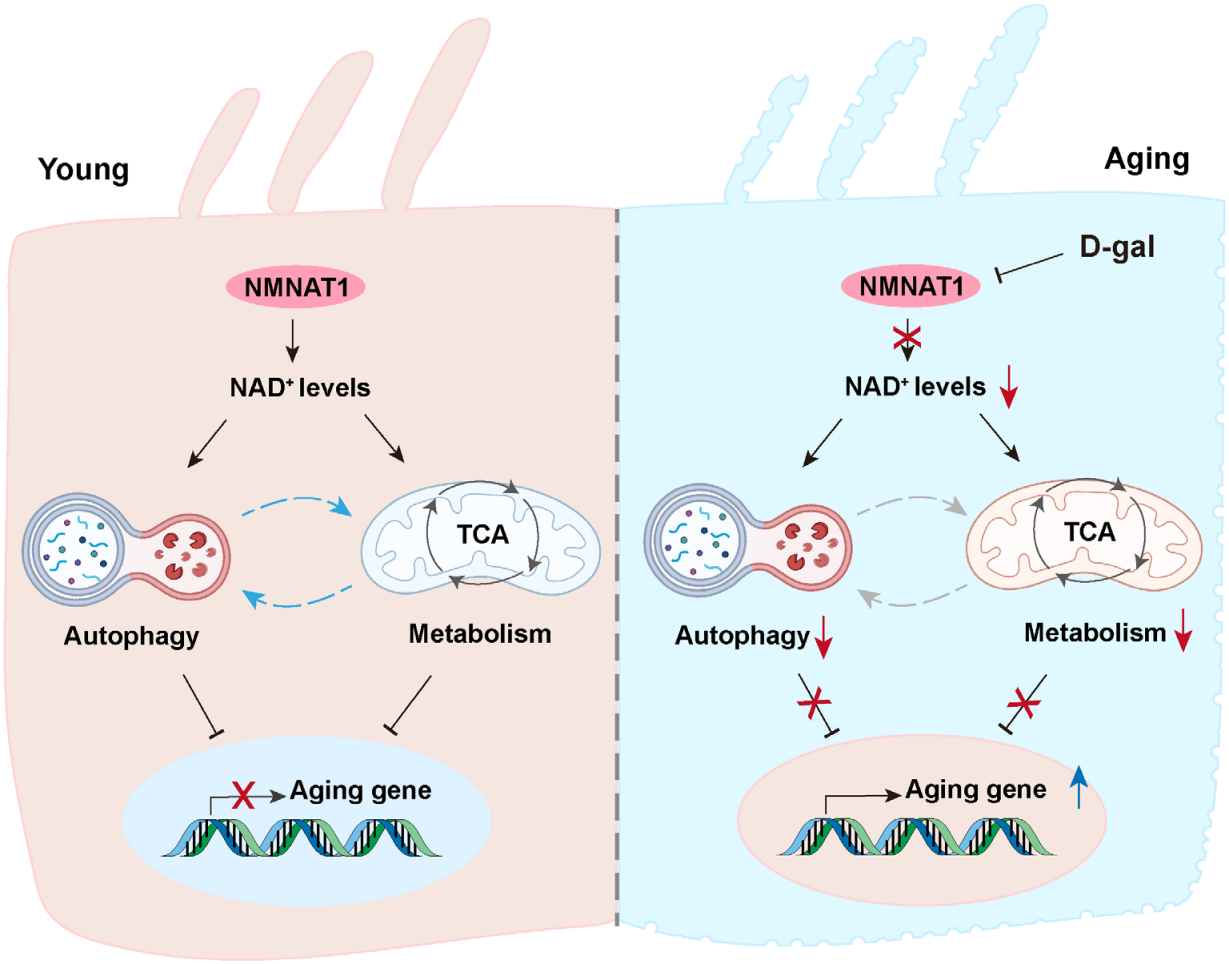
Schematic illustration of the protective role of NMNAT1 during aging. NMNAT1 suppresses the expression of aging‐related genes by facilitating NAD^+^ synthesis, enhancing autophagy, and supporting tricarboxylic acid (TCA) cycle metabolism in young cochlear hair cells. In contrast, D‐galactose (D‐gal) treatment downregulates NMNAT1, thereby impairing autophagy and TCA cycle activity that subsequently facilitates the expression of aging‐related genes in D‐gal‐induced aging hair cells.

However, the interplay between autophagy and metabolism during aging requires further assessment. In this study, we observed reduced autophagy levels and downregulation of NMNAT1 in a D‐gal‐induced aging model, with NMNAT1 overexpression resulting in the activation of autophagy and potentially delaying aging. Additionally, NMNAT1 deletion disrupted the TCA cycle and pentose phosphate pathway (Figure 8). Therefore, our findings offer compelling evidence for the interplay between autophagy and cellular metabolism during aging, identifying potential targets for the diagnosis and treatment of ARHL. However, the mechanisms by which NMNAT1 regulates autophagy and metabolism during aging remain unclear.

## Author Contributions

Yongjie Wei, Wenqing Yang, Han Wu, and Mengdie Kong have contributed equally as first authors to this work. Ye Zhang, Wei Cao, Jianming Yang, and Qiaojun Fang have made substantial contributions to conception, design, funding acquisition, supervision, and writing review and editing. Yongjie Wei, Wenqing Yang, Han Wu, and Mengdie Kong have performed project administration, investigation (execution of most experiments), visualization (figure organization), and writing – original draft preparation, review, and editing. Dachuan Fan, Yuhua Zhang, and Nan Cheng undertook the explants culture, data collection, data curation, and formal analysis. Jiawei Du and Lingna Guo helped with formal analysis, validation, review and editing. Yuyang Li has performed key supplementary experiments. Qian Dai helped perform supplementary experiments and revised the manuscript.

## Funding

The authors have nothing to report.

## Conflicts of Interest

The authors declare no conflicts of interest.

## Supporting information


**Figures S1–S6:** acel70373‐sup‐0001‐FiguresS1‐S6.docx.

**Appendix S1:** acel70373‐sup‐0002‐AppendixS1.pdf.

**Appendix S2:** acel70373‐sup‐0003‐AppendixS2.pdf.

**Table S1:** acel70373‐sup‐0004‐TableS1.xlsx.

**Table S2:** acel70373‐sup‐0005‐TableS2.xlsx.

## Data Availability

The data that supports the findings of this study are available in the Figures S1–S6, Appendices S1 and S2, and Tables S1 and S2 of this article.

## References

[acel70373-bib-0001] Aman, Y. , T. Schmauck‐Medina , M. Hansen , et al. 2021. “Autophagy in Healthy Aging and Disease.” Nature Aging 1, no. 8: 634–650. 34901876 10.1038/s43587-021-00098-4 PMC8659158

[acel70373-bib-0002] Bazard, P. , J. Pineros , R. D. Frisina , et al. 2021. “Cochlear Inflammaging in Relation to Ion Channels and Mitochondrial Functions.” Cells 10, no. 10: 2761. 34685743 10.3390/cells10102761 PMC8534887

[acel70373-bib-0003] Bjedov, I. , H. M. Cocheme , A. Foley , et al. 2020. “Fine‐Tuning Autophagy Maximises Lifespan and Is Associated With Changes in Mitochondrial Gene Expression in Drosophila.” PLoS Genetics 16, no. 11: e1009083. 33253201 10.1371/journal.pgen.1009083 PMC7738165

[acel70373-bib-0004] Castellano, B. M. , A. M. Thelen , O. Moldavski , et al. 2017. “Lysosomal Cholesterol Activates mTORC1 via an SLC38A9‐Niemann‐Pick C1 Signaling Complex.” Science 355, no. 6331: 1306–1311. 28336668 10.1126/science.aag1417 PMC5823611

[acel70373-bib-0006] Covarrubias, A. J. , R. Perrone , A. Grozio , and E. Verdin . 2021. “NAD(+) Metabolism and Its Roles in Cellular Processes During Ageing.” Nature Reviews. Molecular Cell Biology 22, no. 2: 119–141. 33353981 10.1038/s41580-020-00313-x PMC7963035

[acel70373-bib-0007] Davalli, P. , T. Mitic , A. Caporali , A. Lauriola , and D. D'Arca . 2016. “ROS, Cell Senescence, and Novel Molecular Mechanisms in Aging and Age‐Related Diseases.” Oxidative Medicine and Cellular Longevity 2016: 3565127. 27247702 10.1155/2016/3565127 PMC4877482

[acel70373-bib-0008] Debnath, J. , N. Gammoh , and K. M. Ryan . 2023. “Autophagy and Autophagy‐Related Pathways in Cancer.” Nature Reviews. Molecular Cell Biology 24, no. 8: 560–575. 36864290 10.1038/s41580-023-00585-z PMC9980873

[acel70373-bib-0009] Dzau, V. J. , and C. A. Balatbat . 2018. “Health and Societal Implications of Medical and Technological Advances.” Science Translational Medicine 10, no. 463: eaau4778. 30333239 10.1126/scitranslmed.aau4778

[acel70373-bib-0010] Eckert, M. A. , K. C. Harris , H. Lang , et al. 2021. “Translational and Interdisciplinary Insights Into Presbyacusis: A Multidimensional Disease.” Hearing Research 402: 108109. 33189490 10.1016/j.heares.2020.108109 PMC7927149

[acel70373-bib-0011] Fang, E. F. , Y. Hou , S. Lautrup , et al. 2019. “NAD(+) Augmentation Restores Mitophagy and Limits Accelerated Aging in Werner Syndrome.” Nature Communications 10, no. 1: 5284. 10.1038/s41467-019-13172-8 PMC6872719 31754102

[acel70373-bib-0012] Guo, B. , Q. Guo , Z. Wang , et al. 2020. “D‐Galactose‐Induced Oxidative Stress and Mitochondrial Dysfunction in the Cochlear Basilar Membrane: An In Vitro Aging Model.” Biogerontology 21, no. 3: 311–323. 32026209 10.1007/s10522-020-09859-x PMC7196095

[acel70373-bib-0013] He, Z. H. , M. Li , Q. J. Fang , et al. 2021. “FOXG1 Promotes Aging Inner Ear Hair Cell Survival Through Activation of the Autophagy Pathway.” Autophagy 17, no. 12: 4341–4362. 34006186 10.1080/15548627.2021.1916194 PMC8726647

[acel70373-bib-0014] He, Z. H. , S. Pan , H. W. Zheng , Q. J. Fang , K. Hill , and S. H. Sha . 2021. “Treatment With Calcineurin Inhibitor FK506 Attenuates Noise‐Induced Hearing Loss.” Frontiers in Cell and Developmental Biology 9: 648461. 33777956 10.3389/fcell.2021.648461 PMC7994600

[acel70373-bib-0015] Huang, H. , S. Wang , H. Xia , et al. 2024. “Lactate Enhances NMNAT1 Lactylation to Sustain Nuclear NAD(+) Salvage Pathway and Promote Survival of Pancreatic Adenocarcinoma Cells Under Glucose‐Deprived Conditions.” Cancer Letters 588: 216806. 38467179 10.1016/j.canlet.2024.216806

[acel70373-bib-0016] Isgrig, K. , D. S. McDougald , J. Zhu , H. J. Wang , J. Bennett , and W. W. Chien . 2019. “AAV2.7m8 Is a Powerful Viral Vector for Inner Ear Gene Therapy.” Nature Communications 10, no. 1: 427. 10.1038/s41467-018-08243-1 PMC6347594 30683875

[acel70373-bib-0017] Jafari, Z. , B. E. Kolb , and M. H. Mohajerani . 2021. “Age‐Related Hearing Loss and Cognitive Decline: MRI and Cellular Evidence.” Annals of the New York Academy of Sciences 1500, no. 1: 17–33. 34114212 10.1111/nyas.14617

[acel70373-bib-0018] Jayaram, H. N. , P. Kusumanchi , and J. A. Yalowitz . 2011. “NMNAT Expression and Its Relation to NAD Metabolism.” Current Medicinal Chemistry 18, no. 13: 1962–1972. 21517776 10.2174/092986711795590138

[acel70373-bib-0019] Kaushik, S. , I. Tasset , E. Arias , et al. 2021. “Autophagy and the Hallmarks of Aging.” Ageing Research Reviews 72: 101468. 34563704 10.1016/j.arr.2021.101468 PMC8616816

[acel70373-bib-0020] Khawar, M. B. , H. Gao , and W. Li . 2019. “Autophagy and Lipid Metabolism.” Advances in Experimental Medicine and Biology 1206: 359–374. 31776994 10.1007/978-981-15-0602-4_17

[acel70373-bib-0021] Kociszewska, D. , and S. Vlajkovic . 2022. “Age‐Related Hearing Loss: The Link Between Inflammaging, Immunosenescence, and Gut Dysbiosis.” International Journal of Molecular Sciences 23, no. 13: 7348. 35806352 10.3390/ijms23137348 PMC9266910

[acel70373-bib-0022] Lau, C. , C. Dolle , T. I. Gossmann , L. Agledal , M. Niere , and M. Ziegler . 2010. “Isoform‐Specific Targeting and Interaction Domains in Human Nicotinamide Mononucleotide Adenylyltransferases.” Journal of Biological Chemistry 285, no. 24: 18868–18876. 20388704 10.1074/jbc.M110.107631 PMC2881809

[acel70373-bib-0023] Lautrup, S. , D. A. Sinclair , M. P. Mattson , and E. F. Fang . 2019. “NAD(+) in Brain Aging and Neurodegenerative Disorders.” Cell Metabolism 30, no. 4: 630–655. 31577933 10.1016/j.cmet.2019.09.001 PMC6787556

[acel70373-bib-0024] Levine, B. , and G. Kroemer . 2019. “Biological Functions of Autophagy Genes: A Disease Perspective.” Cell 176, no. 1–2: 11–42. 30633901 10.1016/j.cell.2018.09.048 PMC6347410

[acel70373-bib-0025] Lin, P. W. , M. L. Chu , and H. S. Liu . 2021. “Autophagy and Metabolism.” Kaohsiung Journal of Medical Sciences 37, no. 1: 12–19. 33021078 10.1002/kjm2.12299 PMC11896468

[acel70373-bib-0026] Lopez‐Otin, C. , M. A. Blasco , L. Partridge , M. Serrano , and G. Kroemer . 2023. “Hallmarks of Aging: An Expanding Universe.” Cell 186, no. 2: 243–278. 36599349 10.1016/j.cell.2022.11.001

[acel70373-bib-0027] Martinez‐Reyes, I. , and N. S. Chandel . 2020. “Mitochondrial TCA Cycle Metabolites Control Physiology and Disease.” Nature Communications 11, no. 1: 102. 10.1038/s41467-019-13668-3 PMC6941980 31900386

[acel70373-bib-0028] Matias, I. , L. P. Diniz , I. V. Damico , et al. 2022. “Loss of Lamin‐B1 and Defective Nuclear Morphology Are Hallmarks of Astrocyte Senescence In Vitro and in the Aging Human Hippocampus.” Aging Cell 21, no. 1: e13521. 34894056 10.1111/acel.13521 PMC8761005

[acel70373-bib-0029] McReynolds, M. R. , K. Chellappa , and J. A. Baur . 2020. “Age‐Related NAD(+) Decline.” Experimental Gerontology 134: 110888. 32097708 10.1016/j.exger.2020.110888 PMC7442590

[acel70373-bib-0030] Mitchell, S. J. , M. Bernier , M. A. Aon , et al. 2018. “Nicotinamide Improves Aspects of Healthspan, but Not Lifespan, in Mice.” Cell Metabolism 27, no. 3: 667–676. 29514072 10.1016/j.cmet.2018.02.001 PMC5854409

[acel70373-bib-0031] Navas, L. E. , and A. Carnero . 2021. “NAD(+) Metabolism, Stemness, the Immune Response, and Cancer.” Signal Transduction and Targeted Therapy 6, no. 1: 2. 33384409 10.1038/s41392-020-00354-w PMC7775471

[acel70373-bib-0032] Okur, M. N. , B. Mao , R. Kimura , et al. 2020. “Short‐Term NAD(+) Supplementation Prevents Hearing Loss in Mouse Models of Cockayne Syndrome.” NPJ Aging and Mechanisms of Disease 6: 1. 31934345 10.1038/s41514-019-0040-z PMC6946667

[acel70373-bib-0033] Okur, M. N. , B. D. Sahbaz , R. Kimura , et al. 2023. “Long‐Term NAD+ Supplementation Prevents the Progression of Age‐Related Hearing Loss in Mice.” Aging Cell 22, no. 9: e13909. 37395319 10.1111/acel.13909 PMC10497810

[acel70373-bib-0034] Pang, J. , H. Xiong , Y. Ou , et al. 2019. “SIRT1 Protects Cochlear Hair Cell and Delays Age‐Related Hearing Loss via Autophagy.” Neurobiology of Aging 80: 127–137. 31170533 10.1016/j.neurobiolaging.2019.04.003

[acel70373-bib-0035] Ross, S. M. 2021. “Nicotinamide Adenine Dinucleotide (NAD+) Biosynthesis in the Regulation of Metabolism, Aging, and Neurodegeneration.” Holistic Nursing Practice 35, no. 4: 230–232. 34115741 10.1097/HNP.0000000000000461

[acel70373-bib-0036] Rossi, F. , P. C. Geiszler , W. Meng , et al. 2018. “NAD‐Biosynthetic Enzyme NMNAT1 Reduces Early Behavioral Impairment in the Htau Mouse Model of Tauopathy.” Behavioural Brain Research 339: 140–152. 29175372 10.1016/j.bbr.2017.11.030 PMC5769520

[acel70373-bib-0037] Saleh, D. O. , D. F. Mansour , I. M. Hashad , and R. M. Bakeer . 2019. “Effects of Sulforaphane on D‐Galactose‐Induced Liver Aging in Rats: Role of Keap‐1/Nrf‐2 Pathway.” European Journal of Pharmacology 855: 40–49. 31039346 10.1016/j.ejphar.2019.04.043

[acel70373-bib-0038] Schweiger, M. , K. Hennig , F. Lerner , et al. 2001. “Characterization of Recombinant Human Nicotinamide Mononucleotide Adenylyl Transferase (NMNAT), a Nuclear Enzyme Essential for NAD Synthesis.” FEBS Letters 492, no. 1–2: 95–100. 11248244 10.1016/s0014-5793(01)02180-9

[acel70373-bib-0039] Shao, C. , K. Guo , L. Xu , et al. 2021. “Senescence Marker Protein 30 Inhibits Tumor Growth by Reducing HDAC4 Expression in Non‐Small Cell Lung Cancer.” Translational Lung Cancer Research 10, no. 12: 4558–4573. 35070761 10.21037/tlcr-21-982 PMC8743512

[acel70373-bib-0040] Shi, X. , Y. Jiang , A. Kitano , et al. 2021. “Nuclear NAD(+) Homeostasis Governed by NMNAT1 Prevents Apoptosis of Acute Myeloid Leukemia Stem Cells.” Science Advances 7, no. 30: eabf3895. 34290089 10.1126/sciadv.abf3895 PMC8294764

[acel70373-bib-0041] Sokolov, D. , E. R. Sechrest , Y. Wang , C. Nevin , J. Du , and S. Kolandaivelu . 2021. “Nuclear NAD(+)‐Biosynthetic Enzyme NMNAT1 Facilitates Development and Early Survival of Retinal Neurons.” eLife 10: e71185. 34878972 10.7554/eLife.71185 PMC8754432

[acel70373-bib-0042] Tarrago, M. G. , C. C. S. Chini , K. S. Kanamori , et al. 2018. “A Potent and Specific CD38 Inhibitor Ameliorates Age‐Related Metabolic Dysfunction by Reversing Tissue NAD(+) Decline.” Cell Metabolism 27, no. 5: 1081–1095. 29719225 10.1016/j.cmet.2018.03.016 PMC5935140

[acel70373-bib-0043] Tawfik, K. O. , K. Klepper , J. Saliba , and R. A. Friedman . 2020. “Advances in Understanding of Presbycusis.” Journal of Neuroscience Research 98, no. 9: 1685–1697. 30950547 10.1002/jnr.24426

[acel70373-bib-0044] Uchida, Y. , S. Sugiura , Y. Nishita , N. Saji , M. Sone , and H. Ueda . 2019. “Age‐Related Hearing Loss and Cognitive Decline—The Potential Mechanisms Linking the Two.” Auris Nasus Larynx 46, no. 1: 1–9. 30177417 10.1016/j.anl.2018.08.010

[acel70373-bib-0045] Verdin, E. 2015. “NAD(+) in Aging, Metabolism, and Neurodegeneration.” Science 350, no. 6265: 1208–1213. 26785480 10.1126/science.aac4854

[acel70373-bib-0046] Wang, P. , Y. Lu , D. Han , et al. 2019. “Neuroprotection by Nicotinamide Mononucleotide Adenylyltransferase 1 With Involvement of Autophagy in an Aged Rat Model of Transient Cerebral Ischemia and Reperfusion.” Brain Research 1723: 146391. 31421130 10.1016/j.brainres.2019.146391

[acel70373-bib-0047] Wei, Y. , Y. Zhang , W. Cao , et al. 2025. “RONIN/HCF1‐TFEB Axis Protects Against D‐Galactose‐Induced Cochlear Hair Cell Senescence Through Autophagy Activation.” Advanced Science 12: e2407880. 39985193 10.1002/advs.202407880 PMC12362728

[acel70373-bib-0048] Wen, Y. , R. G. Zhai , and M. D. Kim . 2013. “The Role of Autophagy in Nmnat‐Mediated Protection Against Hypoxia‐Induced Dendrite Degeneration.” Molecular and Cellular Neurosciences 52: 140–151. 23159780 10.1016/j.mcn.2012.11.008 PMC3540192

[acel70373-bib-0064] WHO . 2021. World Health Organization. World Report on Hearing, WHO. https://www.who.int/publications/i/item/world‐report‐on‐hearing.

[acel70373-bib-0049] Wolfson, R. L. , L. Chantranupong , G. A. Wyant , et al. 2017. “KICSTOR Recruits GATOR1 to the Lysosome and Is Necessary for Nutrients to Regulate mTORC1.” Nature 543, no. 7645: 438–442. 28199306 10.1038/nature21423 PMC5360989

[acel70373-bib-0050] Wong, S. Q. , A. V. Kumar , J. Mills , and L. R. Lapierre . 2020. “Autophagy in Aging and Longevity.” Human Genetics 139, no. 3: 277–290. 31144030 10.1007/s00439-019-02031-7 PMC6884674

[acel70373-bib-0051] Wu, Y. , Z. Pei , and P. Qu . 2024. “NAD(+)‐A Hub of Energy Metabolism in Heart Failure.” International Journal of Medical Sciences 21, no. 2: 369–375. 38169534 10.7150/ijms.89370 PMC10758143

[acel70373-bib-0052] Xiong, H. , S. Chen , L. Lai , et al. 2019. “Modulation of miR‐34a/SIRT1 Signaling Protects Cochlear Hair Cells Against Oxidative Stress and Delays Age‐Related Hearing Loss Through Coordinated Regulation of Mitophagy and Mitochondrial Biogenesis.” Neurobiology of Aging 79: 30–42. 31026620 10.1016/j.neurobiolaging.2019.03.013

[acel70373-bib-0053] Yang, J. , R. Zhou , and Z. Ma . 2019. “Autophagy and Energy Metabolism.” Advances in Experimental Medicine and Biology 1206: 329–357. 31776993 10.1007/978-981-15-0602-4_16

[acel70373-bib-0054] Yang, W. , X. Zhao , R. Chai , and J. Fan . 2023. “Progress on Mechanisms of Age‐Related Hearing Loss.” Frontiers in Neuroscience 17: 1253574. 37727326 10.3389/fnins.2023.1253574 PMC10505809

[acel70373-bib-0055] Ye, B. , C. Fan , Y. Shen , Q. Wang , H. Hu , and M. Xiang . 2018. “The Antioxidative Role of Autophagy in Hearing Loss.” Frontiers in Neuroscience 12: 1010. 30686976 10.3389/fnins.2018.01010 PMC6333736

[acel70373-bib-0056] Yoshino, J. , K. F. Mills , M. J. Yoon , and S. Imai . 2011. “Nicotinamide Mononucleotide, a Key NAD(+) Intermediate, Treats the Pathophysiology of Diet‐ and Age‐Induced Diabetes in Mice.” Cell Metabolism 14, no. 4: 528–536. 21982712 10.1016/j.cmet.2011.08.014 PMC3204926

[acel70373-bib-0057] Youn, C. K. , Y. Jun , E. R. Jo , and S. I. Cho . 2020. “Age‐Related Hearing Loss in C57BL/6J Mice Is Associated With Mitophagy Impairment in the Central Auditory System.” International Journal of Molecular Sciences 21, no. 19: 7202. 33003463 10.3390/ijms21197202 PMC7584026

[acel70373-bib-0058] Yu, H. , D. Gan , Z. Luo , et al. 2024. “Alpha‐Ketoglutarate Improves Cardiac Insufficiency Through NAD(+)‐SIRT1 Signaling‐Mediated Mitophagy and Ferroptosis in Pressure Overload‐Induced Mice.” Molecular Medicine (Cambridge, Mass) 30, no. 1: 15. 38254035 10.1186/s10020-024-00783-1 PMC10804789

[acel70373-bib-0059] Yuan, H. , X. Wang , K. Hill , et al. 2015. “Autophagy Attenuates Noise‐Induced Hearing Loss by Reducing Oxidative Stress.” Antioxidants & Redox Signaling 22, no. 15: 1308–1324. 25694169 10.1089/ars.2014.6004 PMC4410759

[acel70373-bib-0060] Zhao, C. , Z. Chen , W. Liang , Z. Yang , Z. Du , and S. Gong . 2022. “D‐Galactose‐Induced Accelerated Aging Model on Auditory Cortical Neurons by Regulating Oxidative Stress and Apoptosis In Vitro.” Journal of Nutrition, Health & Aging 26, no. 1: 13–22. 10.1007/s12603-021-1721-4 PMC12275603 35067698

[acel70373-bib-0061] Zhou, J. , S. Y. Chong , A. Lim , et al. 2017. “Changes in Macroautophagy, Chaperone‐Mediated Autophagy, and Mitochondrial Metabolism in Murine Skeletal and Cardiac Muscle During Aging.” Aging (Albany NY) 9, no. 2: 583–599. 28238968 10.18632/aging.101181 PMC5361683

[acel70373-bib-0062] Zhu, Y. , A. G. Lobato , R. G. Zhai , and M. Pinto . 2022. “Human Nmnat1 Promotes Autophagic Clearance of Amyloid Plaques in a Drosophila Model of Alzheimer's Disease.” Frontiers in Aging Neuroscience 14: 852972. 35401143 10.3389/fnagi.2022.852972 PMC8988035

[acel70373-bib-0063] Zhu, Y. , L. Zhang , Y. Sasaki , J. Milbrandt , and J. M. Gidday . 2013. “Protection of Mouse Retinal Ganglion Cell Axons and Soma From Glaucomatous and Ischemic Injury by Cytoplasmic Overexpression of Nmnat1.” Investigative Ophthalmology & Visual Science 54, no. 1: 25–36. 23211826 10.1167/iovs.12-10861 PMC3541947

