# Supplementary material for: NMNAT1 Activates Autophagy to Delay D‐Galactose‐Induced Aging in Cochlear Hair Cells
Source: Aging Cell. 2026 Jan 11;25(2):e70373. doi: 10.1111/acel.70373 (PMC12793064; doi:10.1111/acel.70373)
Supplement: Supplementary file 1 — Figures S1–S6: acel70373‐sup‐0001‐FiguresS1‐S6.docx. [file ACEL-25-e70373-s004.docx]

**Supporting Information**

**Figure S1**


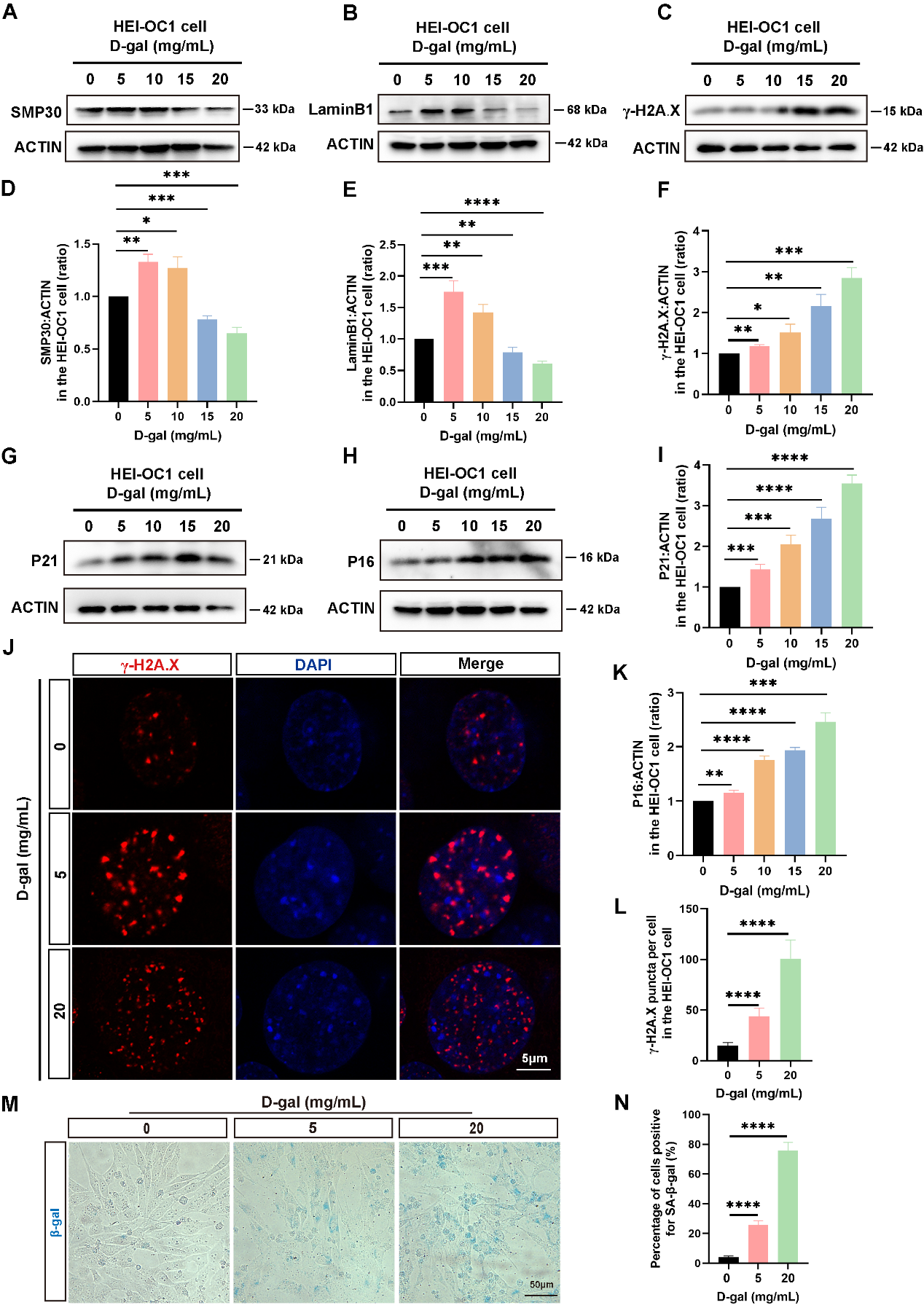


**Figure S1. Aging-related marker expression in HEI-OC1 cells treated with different D-gal concentrations.** (A-C) Western blotting of senescence marker protein-30 (SMP30), Lamin B1, and γ-H2A.X expression in HEI-OC1 cells exposed to 5, 10, 15, and 20 mg/mL D-gal. (D-F) Quantitative analysis of SMP30 (*n* = 3, independent samples), Lamin B1 (*n* = 4, independent samples), and γ-H2A.X (*n* = 3, independent samples) expression in A-C. (G-H) Western blotting of P21 and P16 expression in D-gal treated HEI-OC1 cells. (I) Statistical analysis of P21 expression in G (*n* = 4, independent samples). (J) Immunofluorescence staining demonstrating the number of γ-H2A.X aggregation spots in HEI-OC1 cells exposed to 5 and 20 mg/mL D-gal. (K) Statistical analysis of P16 expression in H (*n* = 3, independent samples). (L) Statistical analysis of γ-H2A.X aggregation spots (*n* = 10; 10 cells from four independent samples), Scale bar: 5 µm. (M) Senescence-associated β-galactosidase (SA-β-gal) staining images in D-gal treated HEI-OC1 cells. (N) Statistical analysis of the percentage of SA-β-gal^+^ cells in M (*n* = 4, independent samples). Scale bar: 50 µm. Statistical significance is indicated as **p* < 0.05, ***p* < 0.01, ****p* < 0.001, and *****p* < 0.0001.

**Figure S2**


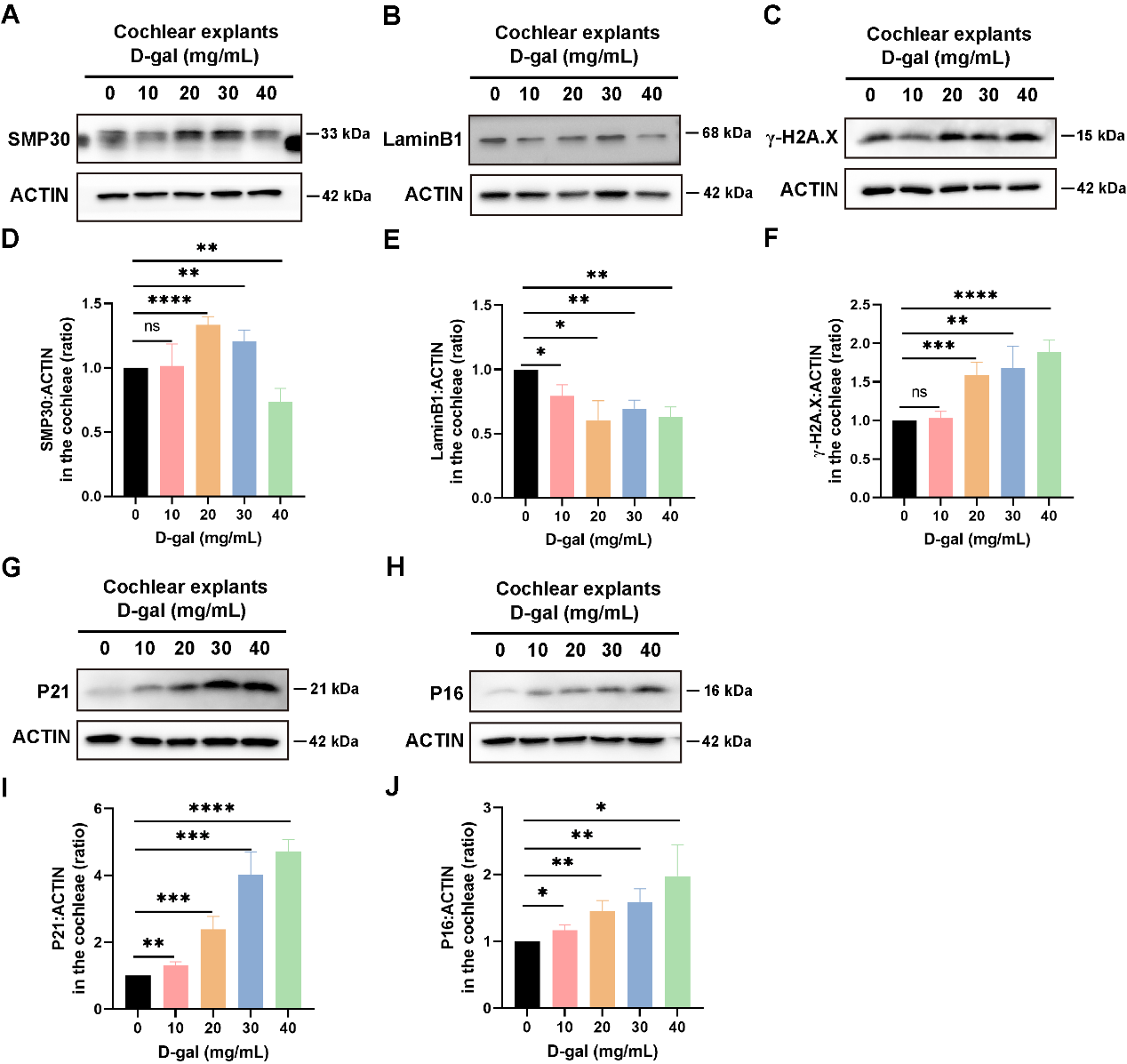


**Figure S2. Aging-related marker expression in cochlear explants exposed to different D-gal concentrations.** (A-C) Western blotting of SMP30, Lamin B1, and γ-H2A.X expression in cochlear explants exposed to 10, 20, 30, and 40 mg/mL D-gal for 72 h. (D-F) Statistical analysis of SMP30 (*n* = 4), Lamin B1 (*n* = 3), and γ-H2A.X (*n* = 4) expression in A-C. (G-H) Western blotting of P21 and P16 expression in D-gal treated cochlear explants. (I-J) Statistical analysis of P21 (*n* = 4) and P16 (*n* = 3) expression in G-H. *n* corresponds to the number of independent samples, each consisting of 12 explants from six mice. Statistical significance is indicated as **p* < 0.05, ***p* < 0.01, ****p* < 0.001, and *****p* < 0.0001.

**Figure S3**


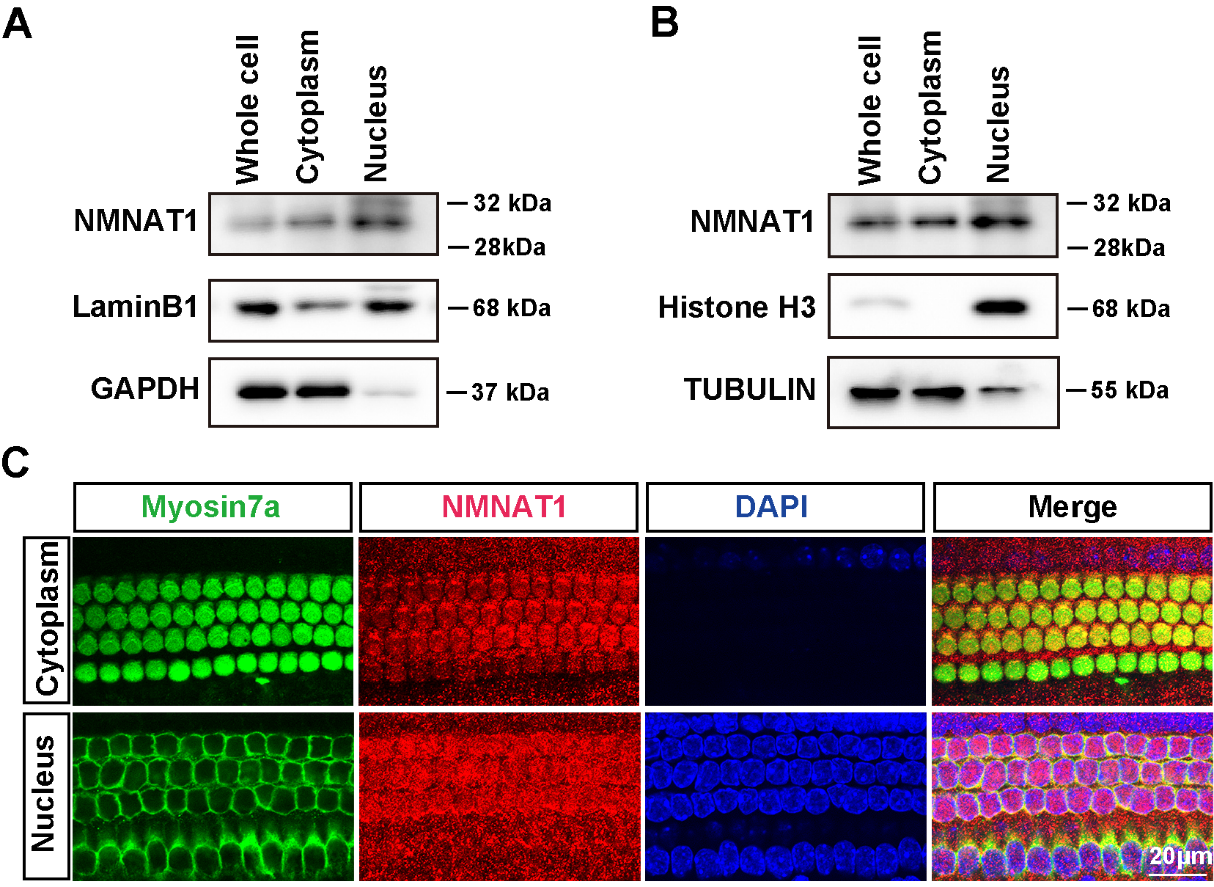


**Figure S3. Subcellular localization of *Nmnat1* in HEI-OC1 cells and cochlear hair cells.** (A-B) Western blotting analysis of *Nmnat1* in the cytoplasmic and nucleus fractions of HEI-OC1 cells. (C) Immunofluorescence staining demonstrating the subcellular localization of *Nmnat1* in cochlear hair cells, Scale bar: 20 µm.

**Figure S4**


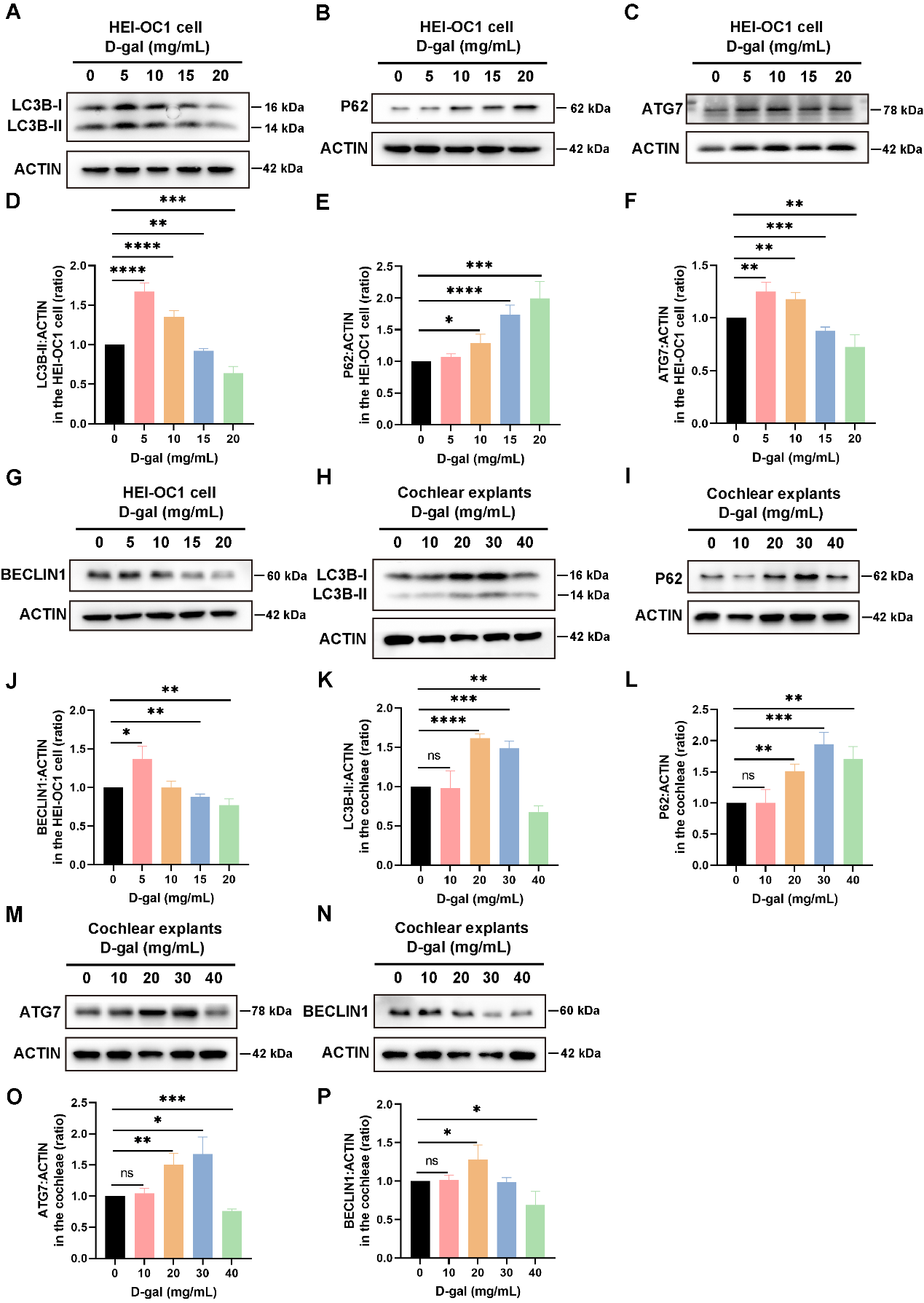


**Figure S4. Autophagy-related marker expression in HEI-OC1 cells and cochlear explants exposed to different concentrations of D-gal.** (A-C, G) Western blotting of LC3B-II, P62, ATG7, and BECLIN1 expression in HEI-OC1 cells treated with different concentrations of D-gal. (D-F, J) Statistical analysis of LC3B-II (*n* = 4), P62 (*n* = 4), ATG7 (*n* = 4), and BECLIN1 (*n* = 3) expression in A-C and G. (H-I and M-N) Western blotting of LC3B-II, P62, ATG7, and BECLIN1 expression in cochlear explants treated with different concentrations of D-gal. (K-L and O-P) Statistical analysis of LC3B-II (*n* = 3), P62 (*n* = 3), ATG7 (*n* = 3), and BECLIN1 (*n* = 4) expression in H-I and M-N. *n* corresponds to the number of independent samples. Statistical significance is indicated as **p* < 0.05, ***p* < 0.01, ****p* < 0.001, *****p* < 0.0001, and ns: no significant difference.

**Figure S5**


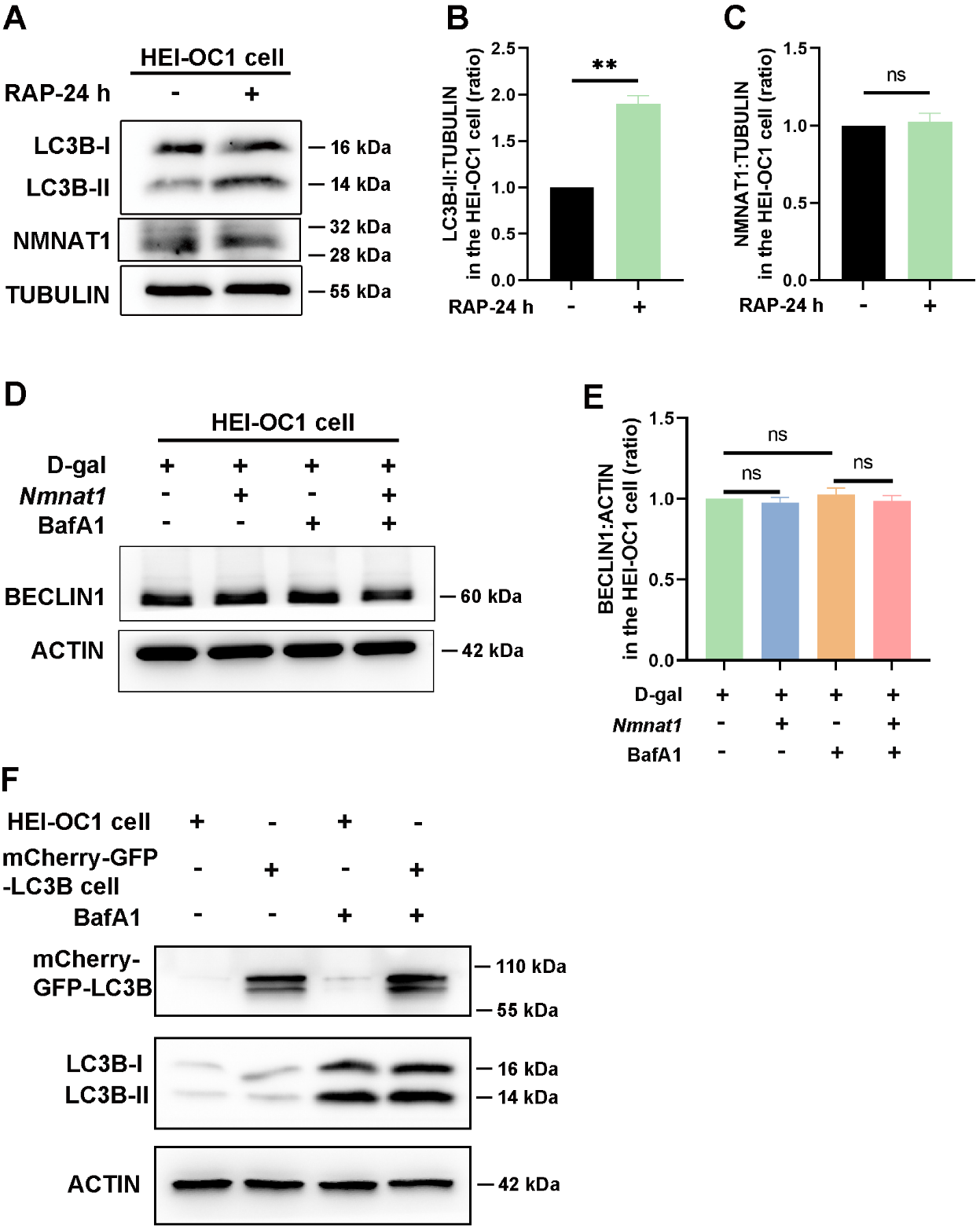


**Figure S5.** **The expression level of NMNAT1 remained unaltered following autophagy activation, and BECLIN1 expression was unaffected by NMNAT1 overexpression in D-gal-treated aging HEI-OC1 cells.** (A) Western blotting of LC3B-II and NMNAT1 expression in HEI-OC1 cells exposed to 100 nM rapamycin for 24 h. (B) Statistical analysis of LC3B-II expression in A (*n* = 3). (C) Statistical analysis of NMNAT1 expression in A (*n* = 3). (D) Western blotting of BECLIN1 in *Nmnat1*-overexpressing HEI-OC1 cells. (E) Statistical analysis of BECLIN1 expression in D (*n* = 4). (F) Western blot analysis of mCherry-GFP-LC3B and LC3B in HEI-OC1 cells and mCherry-GFP-LC3B cell line. *n* corresponds to the number of independent samples. Statistical significance is indicated as ***p* < 0.01, and ns: no significant difference.

**Figure S6**


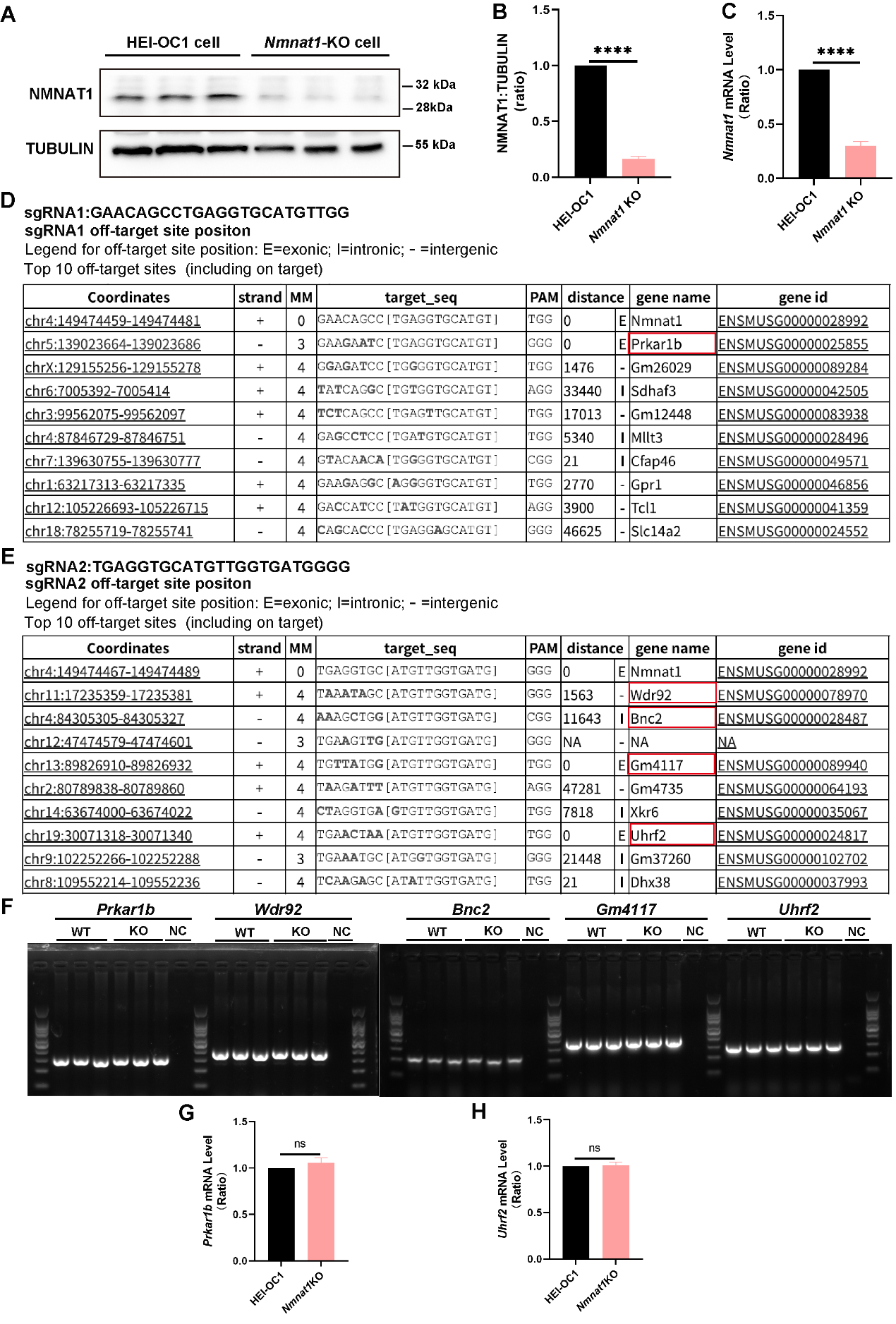


**Figure S6. Verification of knockout (KO) efficiency and off-target effects in *Nmnat1*-KO cells.** (A) Western blotting of NMNAT1 expression in HEI-OC1 cells and *Nmnat1*-KO cells. (B) Statistical analysis of NMNAT1 expression in A (*n* = 3, independent samples). (C) The expression level of *Nmnat1* mRNA is detected in HEI-OC1 and *Nmnat1*-KO cells by reverse transcription quantitative polymerase chain reaction (PCR) (*n* = 3, independent samples). (D-E) The off-target sites of single-guide RNA 1 (sgRNA1) and sgRNA2 are predicted using the CCTop-CRISRP/Cas9 target online predictor website. (F-H) The DNA and RNA levels of potential off-target sites of sgRNA1 and sgRNA2 are detected in HEI-OC1 and *Nmnat1*-KO cells using PCR and q-PCR (*n* = 3, independent samples). WT: HEI-OC1 cells，KO: *Nmnat1*-KO cells, and NC: negative control. Statistical significance is indicated as *****p* < 0.0001, and ns: no significant difference.
