# Supplementary material for: NMNAT1 Activates Autophagy to Delay D‐Galactose‐Induced Aging in Cochlear Hair Cells
Source: Aging Cell. 2026 Jan 11;25(2):e70373. doi: 10.1111/acel.70373 (PMC12793064; doi:10.1111/acel.70373)
Supplement: Supplementary file 2 — Appendix S1: acel70373‐sup‐0002‐AppendixS1.pdf. [file ACEL-25-e70373-s005.pdf]

Results for unnamed

Download full results file here.
Download sgRNAs target sites as fasta file here.
Visualize sgRNA target sites in the Ensembl genome browser.
Visualize sgRNA target sites in the UCSC genome browser.

Detailed results

Species:	Mouse (Mus musculus GRCm38/mm10)
Input:	GAACAGCCTGAGGTGCATGTTGG

PAM:	NGG
Target site length:	20
Target site 5' limitation:	NN
Target site 3' limitation:	NN
Core length:	12
Core MM:	2
Total MM:	4

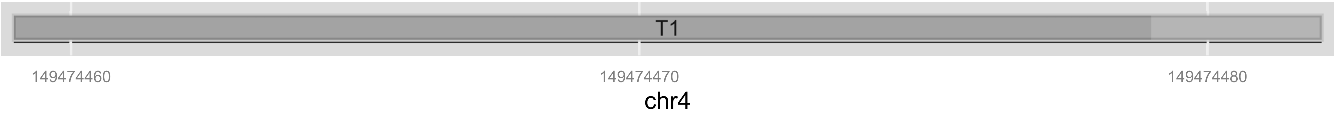

Legend for off-target site positon: E = exonic; I = intronic; - = intergenic
Legend for the CRISPRater score: LOW efficacy (score<0.56); MEDIUM efficacy (0.56<=score<=0.74); HIGH efficacy (score>0.74)

Oligo pair with 5' substitution fwd: TAGgACAGCCTGAGGTGCATGT rev: AAACACATGCACCTCAGGCTGT

Top 20 offtarget sites out of 93 (including on target; for full list see xls file)

Coordinates	strand	MM	target_seq	PAM	distance		gene name	gene id
chr4:149474459-149474481	+	0	GAACAGCC[TGAGGTGCATGT]	TGG	0	E	Nmnat1	ENSMUSG00000028992
chr5:139023664-139023686	-	3	GAAGAATC[TGAGGTGCATGT]	GGG	0	E	Prkar1b	ENSMUSG00000025855
chrX:129155256-129155278	+	4	GGAGATCC[TGGGGTGCATGT]	TGG	1476	-	Gm26029	ENSMUSG00000089284
chr6:7005392-7005414	+	4	TATCAGGC[TGTGGTGCATGT]	AGG	33440	I	Sdhaf3	ENSMUSG00000042505
chr3:99562075-99562097	+	4	TCTCAGCC[TGAGTTGCATGT]	TGG	17013	-	Gm12448	ENSMUSG00000083938
chr4:87846729-87846751	-	4	GAGCCTCC[TGATGTGCATGT]	TGG	5340	I	Mllt3	ENSMUSG00000028496
chr7:139630755-139630777	-	4	GTACAACA[TGGGGTGCATGT]	CGG	21	I	Cfap46	ENSMUSG00000049571
chr1:63217313-63217335	+	4	GAAGAGGC[AGGGGTGCATGT]	TGG	2770	-	Gpr1	ENSMUSG00000046856
chr12:105226693-105226715	+	4	GACCATCC[TATGGTGCATGT]	AGG	3900	-	Tcl1	ENSMUSG00000041359
chr18:78255719-78255741	-	4	CAGCACCC[TGAGGAGCATGT]	GGG	46625	-	Slc14a2	ENSMUSG00000024552
chr12:71096526-71096548	-	4	CAACAGTC[AGATGTGCATGT]	GGG	953	I	Arid4a	ENSMUSG00000048118
chr3:8928255-8928277	-	4	GTACAGTC[AGATGTGCATGT]	GGG	0	E	Tpd52	ENSMUSG00000027506
chr16:33018288-33018310	-	4	GAACTTCC[AGATGTGCATGT]	TGG	1131	I	Iqcg	ENSMUSG00000035578
chr3:17284745-17284767	+	4	GAGCAGCA[TTTGGTGCATGT]	TGG	13951	-	Gm6350	ENSMUSG00000103351
chr8:16167885-16167907	+	4	GTACAGAC[TCATGTGCATGT]	AGG	850	I	Mir3106	ENSMUSG00000093142
chr17:68624989-68625011	+	4	AAACAGGC[AGAGTTGCATGT]	GGG	5174	I	L3mbtl4	ENSMUSG00000041565
