# Supplementary material for: NMNAT1 Activates Autophagy to Delay D‐Galactose‐Induced Aging in Cochlear Hair Cells
Source: Aging Cell. 2026 Jan 11;25(2):e70373. doi: 10.1111/acel.70373 (PMC12793064; doi:10.1111/acel.70373)
Supplement: Supplementary file 3 — Appendix S2: acel70373‐sup‐0003‐AppendixS2.pdf. [file ACEL-25-e70373-s003.pdf]

# Results for unnamed

Download full results file here.
Download sgRNAs target sites as fasta file here.
Visualize sgRNA target sites in the Ensembl genome browser.
Visualize sgRNA target sites in the UCSC genome browser.

## Detailed results

Species: Mouse (Mus musculus GRCm38/mm10)
Input: TGAGGTGCATGTTGGTGATGGGG

PAM: NGG
Target site length: 20
Target site 5' limitation: NN
Target site 3' limitation: NN
Core length: 12
Core MM: 2
Total MM: 4

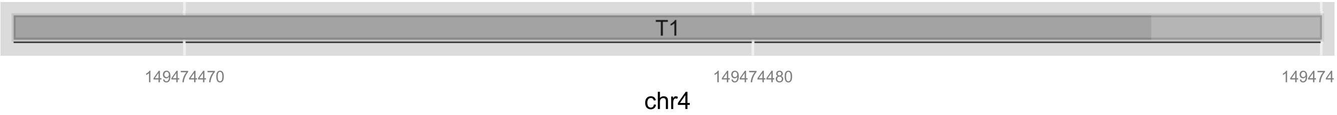


Legend for off-target site positon: E = exonic; I = intronic; - = intergenic
Legend for the CRISPRater score: LOW efficacy (score<0.56); MEDIUM efficacy (0.56<=score<=0.74); HIGH efficacy (score>0.74)

[illegible]

| Coordinates | strand | MM | target_seq | PAM | distance | | gene name | gene id |
|---|---|---|---|---|---|---|---|---|
| chr4:149474467-149474489 | + | 0 | TGAGGTGC [ATGTTGGTGATG] | GGG | 0 | E | Nmnat1 | ENSMUSG00000028992 |
| chr11:17235359-17235381 | + | 4 | TAAATAGC [ATGTTGGTGATG] | GGG | 1563 | - | Wdr92 | ENSMUSG00000078970 |
| chr4:84305305-84305327 | - | 4 | AAAGCTGG [ATGTTGGTGATG] | CGG | 11643 | I | Bnc2 | ENSMUSG00000028487 |
| chr12:47474579-47474601 | - | 3 | TGAAGTTG [ATGTTGGTGATG] | GGG | NA | - | NA | NA |
| chr13:89826910-89826932 | + | 4 | TGTTATGG [ATGTTGGTGATG] | TGG | 0 | E | Gm4117 | ENSMUSG00000089940 |
| chr2:80789838-80789860 | + | 4 | TAAGATTT [ATGTTGGTGATG] | AGG | 47281 | - | Gm4735 | ENSMUSG00000064193 |
| chr14:63674000-63674022 | - | 4 | CTAGGTGA [GTGTTGGTGATG] | TGG | 7818 | I | Xkr6 | ENSMUSG00000035067 |
| chr19:30071318-30071340 | + | 4 | TGAACTAA [ATGTTGGTGATG] | TGG | 0 | E | Uhrf2 | ENSMUSG00000024817 |
| chr9:102252266-102252288 | - | 3 | TGAAATGC [ATGGTGGTGATG] | GGG | 21448 | I | Gm37260 | ENSMUSG00000102702 |
| chr8:109552214-109552236 | - | 4 | TCAAGAGC [ATATTGGTGATG] | TGG | 21 | I | Dhx38 | ENSMUSG00000037993 |
| chr4:92418397-92418419 | + | 4 | TGTGGAGT [CTGTTGGTGATG] | AGG | 45049 | - | Gm12638 | ENSMUSG00000083296 |
| chr4:106443787-106443809 | - | 3 | AGAGATGC [ATGTAGGTGATG] | GGG | 229 | I | Pcsk9 | ENSMUSG00000044254 |
| chr1:120084165-120084187 | + | 4 | TGAGCAGT [GTGTTGGTGATG] | GGG | 10091 | - | Tmem37 | ENSMUSG00000050777 |
| chr2:130379297-130379319 | + | 4 | TGTTCTGC [ATGATGGTGATG] | TGG | 5564 | - | Gm14044 | ENSMUSG00000083483 |
| chr11:8571319-8571341 | - | 3 | TGAGGTCT [ATATTGGTGATG] | AGG | 7742 | I | Tns3 | ENSMUSG00000020422 |
| chr15:22264184-22264206 | - | 4 | AAAGGTGA [ATGCTGGTGATG] | AGG | 9864 | - | Gm27525 | ENSMUSG00000098700 |
| chr14:72532492-72532514 | - | 4 | TGGGCAGC [ATGGTGGTGATG] | GGG | 5432 | - | Fndc3a | ENSMUSG00000033487 |
